# The *Leishmania* ARL-1 and Golgi Traffic

**DOI:** 10.1371/journal.pone.0001620

**Published:** 2008-02-20

**Authors:** Annelise Sahin, Benoît Espiau, Emmanuel Tetaud, Armelle Cuvillier, Lydia Lartigue, Audrey Ambit, Derrick R. Robinson, Gilles Merlin

**Affiliations:** Laboratoire de Microbiologie Cellulaire et Moléculaire et Pathogénicité, UMR CNRS 5234, Université Bordeaux 2, Bordeaux, France; Centre for DNA Fingerprinting and Diagnostics, India

## Abstract

We present here the characterisation of the *Leishmania* small G protein ADP-Ribosylation Factor-Like protein 1 (ARL-1). The ARL-1 gene is present in one copy per haploid genome and conserved among trypanosomatids. It encodes a protein of 20 kDa, which is equally expressed in the insect promastigote and mammalian amastigote forms of the parasite. ARL-1 localises to the Trans-Golgi Network (TGN); N-terminal myristoylation is essential for TGN localisation. *In vivo* expression of the *Ld*ARL-1/Q74L and *Ld*ARL-1/T51N mutants (GTP- and GDP-bound blocked forms respectively) shows that GDP/GTP cycling occurs entirely within the TGN. This is contrary to previous reports in yeast and mammals, where the mutant empty form devoid of nucleotide has been considered as the GDP-blocked form. The dominant-negative empty form mutant *Ld*ARL-1/T34N inhibits endocytosis and intracellular trafficking from the TGN to the Lysosome/Multivesicular Tubule and to the acidocalcisomes; these defects are probably related to a mislocalisation of the GRIP domain-containing vesicle tethering factors which cannot be recruited to the TGN by the cytoplasmic *Ld*ARL-1/T34N. Thus, besides the functional characterization of a new mutant and a better understanding of ARL-1 GDP/GTP cycling, this work shows that *Leishmania* ARL-1 is a key component of an essential pathway worth future study.

## Introduction

*Leishmania sp* are flagellated trypanosomatid parasites responsible for widespread diseases in tropical and subtropical countries (http://www.who.int/tdr/diseases/leish/default.htm). The parasite alternates between a flagellated extracellular form in the insect guts and an aflagellated intracellular form living in the parasitophorous vacuoles of mammalian macrophages. Such particularities and the evolutionary distance make it likely that there are sufficient differences in the biological pathways between parasites and hosts to find new parasite-specific drug targets. This is seriously needed due to the limited choice of available treatments, which are old, and the spreading of drug resistance. Basic research, accompanied by the recent publication of the complete genome sequence of several trypanosomatid species, including *Leishmania* [1], [2], may help to find new approaches to control these diseases.

Intracellular traffic is an essential process in all living organisms. In humans, a number of severe diseases are caused by trafficking deficiencies [3]. It is not absurd to expect to find within such complex machinery a parasite-specific step that is exploitable to impair parasite traffic and viability. To our knowledge, such an achievement has not yet been reached, and it is much too early to know whether and when therapeutic applications may arise. However, there are already parasite-specific pathways candidates, for example the now well studied trafficking of GPI-anchored proteins in trypanosomes [4].

Vesicles represent the main tool of intracellular traffic and a great number of proteins are involved in their assembly, mobility and disassembly, including notably small G proteins, like members of the ARF/ARL (ADP-Ribosylation Factor/ADP-Ribosylation factor-Like) family [5]–[8]. There are 5 or 6 different ARFs and about a dozen ARLs among this family, depending on the species. The most studied are ARF-1 and ARF-6 with orthologues in yeast, plants, mammals [9], and even protozoa like *Leishmania* [10] and *Trypanosoma* [11], [12]. ARL-1 has recently received much attention and orthologues have been studied in several species. ARL-1 has been localised to the trans-Golgi network (TGN) in yeast, mammals [13], [14] and *T. brucei* [15]. Essential in *Drosophila* [16], ARL-1 maintains the integrity of the Golgi apparatus [15], [17], [18], and controls the vesicle traffic and the vacuole formation in yeast [19]–[21]. As for most other members of the family, myristoylation of its N-terminus is essential for its localisation and function [15], [19], [22], [23].

Being a G protein, ARL-1 cycles between GDP- and GTP-bound forms. In yeast, a nucleotide exchange factor, Ysl2p [24], and a GAP (GTPase-activating protein), Gcs1p [25], have been characterised; in mammals, only a yet uncharacterised ARF-GAP has been described [26]. Several effectors have been shown to interact with the GTP-bound form of ARL-1 [14], [18], 27, particularly the GRIP domain of several golgins/tethering factors. In the current model, such interactions allow the recruitment of these tethering factors to TGN membranes, which are essential for the vesicular traffic between the Golgi apparatus and endosomes [28]–[32]. This is probably how ARL-1 participates to the Golgi apparatus maintenance and vesicular traffic.

Recently, remarkable studies with the orthologue *Tb*ARL-1 have been reported in *Trypanosoma brucei*. *Tb*ARL-1 is only expressed in the bloodstream forms, where it is associated with the Golgi apparatus; RNAi experiments showed that *Tb*ARL-1 is essential for viability, Golgi apparatus maintenance and exocytosis in bloodstream forms, but has no effect in insect forms [15]. As *Leishmania*, this organism belongs to the *Trypanosomatidae* family. However, the two genus diverged possibly more than 100 million years ago [33] and they present many different features. To cite a few, the percentage of G/C in the *T. brucei* genome is lower than in *L. major* (41% versus 59.7% respectively) [34]; RNA interference (RNAi) is functional in *T. brucei* but impossible in *Leishmania* and concerning their life cycle, contrary to *Leishmania*, both *T. brucei* insect and mammalian (or bloodstream) forms are flagellated and extracellular, which has important physiological consequences.

We present here the characterisation of *Ld*ARL-1 in *Leishmania*. Contrary to *T. brucei*, *Ld*ARL-1 is expressed in both the insect and mammalian forms of the parasite. Design of a new mutant type, *Ld*ARL-1/T51N, corresponding to the GDP-bound blocked form, revealed that *Ld*ARL-1 cycles entirely within the TGN. This is contrary to previous data obtained in yeast and mammals with another mutant wrongly considered as the GDP-bound blocked form,. Expression of the dominant-negative mutant *Ld*ARL-1/T34N had severe inhibitory effects on intracellular traffic, showing *Ld*ARL-1 involvement in the control of endocytosis, in intracellular trafficking from the TGN to the Lysosome/MVT (Multivesicular tubule) and the acidocalcisomes, and in the TGN targeting of the tethering factor pGRIP-2, a yet uncharacterised *Leishmania* GRIP domain-containing protein.

## Results

### Identification of the *L. donovani* ARL-1 gene

Compilation of the first two consensus motifs of the GTP-binding site (GLDXAGKT, WDXGGQ) of human (*Hs*)ARFs (Swissprot accession numbers: ARF-1, P32889; ARF-3, P16587; ARF-4, P18085; ARF-5, P26437; ARF-6, P26438) and ARLs (ARL-1, P40616; ARL-2, P36404; ARL-3, P36405; ARL-4, P40617) allowed the design of two degenerated oligonucleotides (G019/G020) and the PCR-amplification of 144 bp fragments from *L. donovani* LSB-51.1 genomic DNA. Fragment 4020 showed 70% identity with *Hs*ARL-1 and produced, when used as probe on Southern blots with *L. donovani* genomic DNA, a band pattern compatible with the existence of only one gene (not shown). Subsequent screening of the *L. donovani* LSB 51.1 genomic cosmid library allowed the isolation of a cosmid containing the entire *Ld*ARL-1 ORF (561 bp); this region was sequenced over 1896 bp (Genbank AF187855 and AAF29899). Upstream of the ATG codon, a 34 bp long polypyrimidine tract (−101 to −68) followed by 5 AG dinucleotides (potential spliced-leader attachment sites) [35] ascertained the potential functionality of this gene.

**Figure 1:**
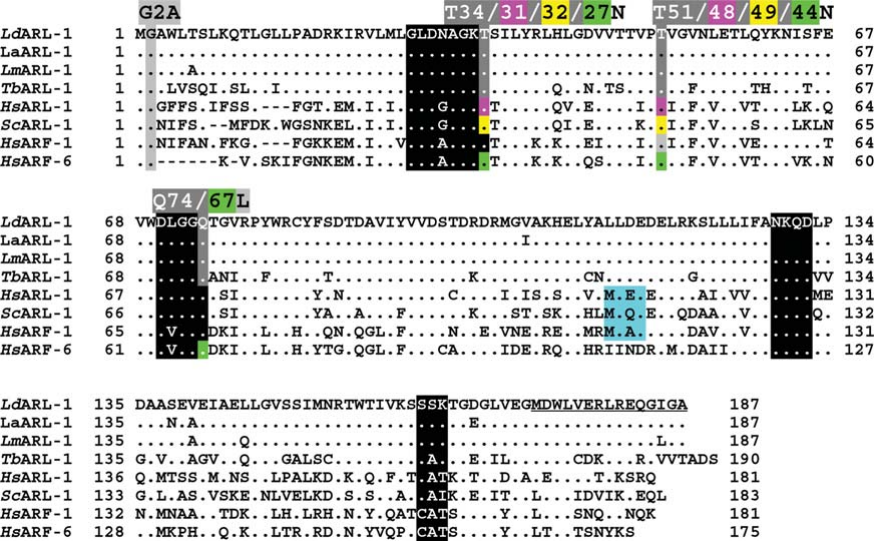
Sequence analysis of ARL-1/ARF-1 of various species. Consensus motives for the GDP-GTP binding site are boxed in black. Mutated and amino acids of interest are boxed in grey. The sequence of the C-terminal peptide synthesised for the production of the rabbit immune serum is underlined. Databanks accession numbers: *Ld*ARL-1, Genbank AF187855 and AAF29899; *La*ARL-1, GenBank XXX; *Lm*ARL-1, GeneDB LmjF17.0070 (http://www.genedb.org/ *)*; *Tb*ARL-1, Genbank AAX70381, GeneDB Tb927.7.6230, previously Tb07.2F2.550; *Hs*ARL-1, Swissprot P40616; ScARL-1: Genbank S46035; *Hs*ARF-1: Swissprot P32889; *Hs*ARF-6: Genbank NP_001654.

The predicted ARL-1 protein, which contains 187 amino acids (20 833 Da, pI 5.24), displays 55% identity with the human *Hs*ARL-1, 52% with yeast *Sc*ARL-1 and 50% with human *Hs*ARF-1 (Fig 1). As for all ARFs/ARLs, the Glycine at position 2 is a potential myristoylation site. The first three consensus motifs of the GDP/GTP binding site (GxxxxGKT, DxxG, and NxxD) [36] are present (Fig 1) while the fourth one (C/SA/xx) is less conserved as often observed for ARFs/ARLs. The ability of the recombinant *Ld*ARL-1 to bind GTPγS has been described elsewhere [37].

### The ARL-1 gene in trypanosomatids

ARL-1 orthologue genes exist in one copy per haploid genome in the parasites genome database (GeneDB: http://www.genedb.org/ *)* for *L. major* (LmjF17.0070, 187 aa, 98% identity) (Fig 1), *L. infantum* (LinJ17.0080, 100% identity), *L. brasiliensis* (LbrM17_V2.0080, 189 aa, 93% identity) and *T. brucei* (Tb927.7.6230, previously Tb07.2F2.550, annotated as *Tb*ARF-3, 190 aa, 72% identity, [15]). After PCR-amplification, we determined the sequence of *L. amazonensis La*ARL-1, which has 4 different amino acids compared to *L. donovani* and the changes are conservative (Fig 1). In the case of *T. cruzi*, there are two entries: Tc00.1047053506513.60 with a similar length (190 aa) to *Ld*ARL-1 and Tc00.1047053508919.60 with a 101 aa N-terminal extension; the ARL-1 part of the second entry hass 3 different aa from the first one and they both share 74% identity with *Ld*ARL-1. Fig S1 shows the gene organization of the ARL-1 region in the genomes of these trypanosomatids.

### *In vivo* expression and subcellular localisation of ARL-1

**Figure 2:**
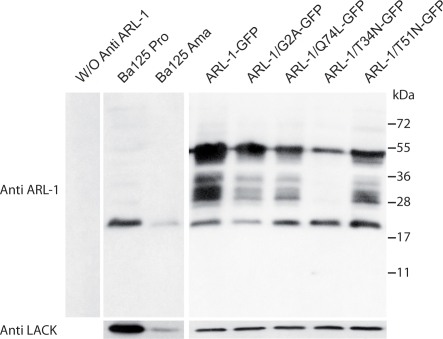
* E*xpression of *Ld*ARL-1 and mutants in *L. amazonensis.* Extracts of 3.10^6^ exponentially growing *L. amazonensis* promastigotes (about 3 µg of proteins) were submitted to western blot analysis using the rabbit anti-*Ld*ARL-1 C-terminus immune serum (1∶5000 dilution); color was developed with an anti-rabbit IgG peroxidase conjugate (1∶10000 dilution) and an ECL revelation kit (Amersham). Internal standard: 37 kDa LACK antigen. Left, negative control without anti-LdARL-1 antiserum. Additional bands are revealed when the GFP-fused proteins are expressed, probably due to partial degradation when the cells were lysed, in spite of the presence of antiproteases (see Methods).

A rabbit immune serum raised against the last 15 aa of *Ld*ARL-1 (underlined in Fig 1) allowed the detection of a unique 20 kDa band in promastigote and amastigote extracts of two strains of *L. amazonensis*, the BA125 strain (Fig. 2) and the LV79 strain (not shown). The expression of ARL-1 was similar in both forms of the parasite.

Untagged or GFP-fused *Ld*ARL-1 variants were expressed in *L. amazonensis* promastigotes using the pTEX [38] or the pNUS-GFPcH [39] vectors, respectively, both leaving the *Ld*ARL-1 N-terminus free. These vectors remain episomal and allow the expression of exogenous proteins. The abundance of the exogenous protein depends on the episome copy number, which can be manipulated within certain limits by the selective antibiotic concentration in the culture medium, and might also vary from cell to cell after unequal partitioning at mitosis. In this study, identical data were obtained with the transfectants expressing untagged or GFP-fused proteins.

Western blot analyses confirmed that the transfectants were expressing the proteins of interest (Fig 2). The endogenous 20 kDa *La*ARL-1 and a 48 kDa protein corresponding to *Ld*ARL-1-GFP were observed. Although *Ld*ARL-1 and *Ld*ARL-1-GFP were expressed at a higher level than the endogenous *La*ARL-1, this had no significant effect on the *in vitro* growth rate of the transgenic cell lines compared to parental cells (Fig S2).

**Figure 3:**
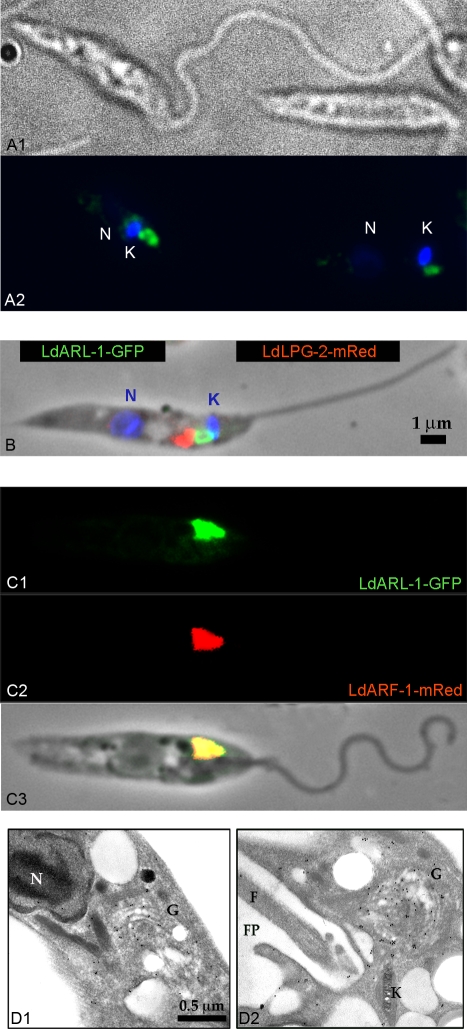
* Ld*ARL-1 subcellular localisation in *L. amazonensis* promastigotes. A. IF on fixed cells: A1, phase contrast; A2, co-labelling with DAPI (blue) and FITC-anti rabbit-IgG plus anti-*Ld*ARL-1 rabbit immune serum (green). B. IF on fixed cells co-transformed with pNUS-*Ld*ARL-1-GFPcH (green) and pNUS-mREDnD-*Ld*LPG-2 (red) plus DAPI staining (blue). C. IF on fixed cells co-transformed with pNUS-*Ld*ARL-1-GFPcH (C1, green) plus pNUS-*Ld*ARF-1-mRedcD (C2, red), overlay (C3). D. EM of cells transformed with pNUS-*Ld*ARL-1-GFP and incubated with anti-*Ld*ARL-1 immune serum. C1 and C2 are sections of two different cells. Black dots (10 nm gold particles) show the *Ld*ARL-1 immunoreactive spots. N: nucleus; G: Golgi apparatus; K: kinetoplast; F: flagellum; FP: flagellar pocket.

**Figure 4:**
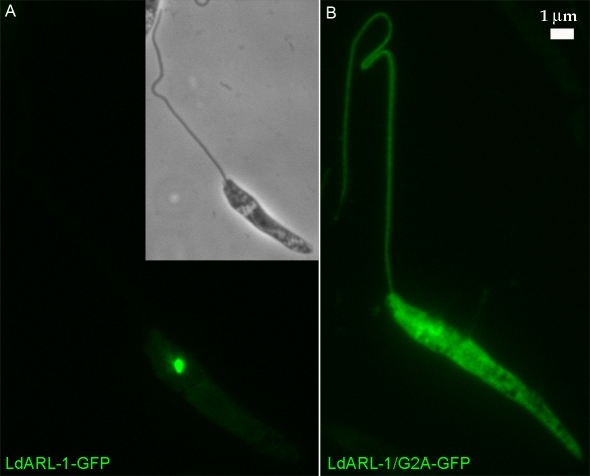
Localisation of the non-myristoylated *Ld*ARL-1. Fixed *L. amazonensis* promastigotes transformed with: A, pNUS-*Ld*ARL-1-GFPcH (green); B, pNUS-*Ld*ARL-1/G2A-GFPcH (green).

Indirect immunofluorescence microscopy observations with the anti-*Ld*ARL-1 antiserum revealed a spot in the vicinity of the kinetoplast and the flagellar pocket (Fig 3A), strongly reminiscent of the TGN localization reported for *Ld*ARF-1-GFP [10]. Promastigotes expressing *Ld*ARL-1-GFP showed the same localisation (Fig 3B, 4A); the green GFP signal was adjacent to the red one of mRed-*Ld*Lpg-2 signal, a Golgi cisternae marker [40] (Fig 3B) and co-localised with *Ld*ARF-1-mRed [10] (Fig 3C). Electron microscopy confirmed the *Ld*ARL-1 TGN localisation (Fig 3D). In the ARL-1 sequence of *L. donovani*, *L. amazonensis*, *L. major* and *T. brucei*, the Golgi localisation signal MXXE [41] present in *Hs*ARL-1, *Sc*ARL-1 and *Hs*ARF-1 (Fig 1) is replaced by LXXE, likely functional according to our IF (immunofluorescence) results.

### *Ld*ARL-1 N-myristoylation

N-myristoylation is important for ARFs membrane binding and biological activity [23]. The *Ld*ARL-1 Glycine 2 (Fig 1) is a potential myristoylation site [5], [42]. When both *Ld*ARL-1 and *L. major* N-myristoyltransferase were coexpressed in *E. coli*, recombinant *Ld*ARL-1 could indeed be myristoylated [43]. To test the biological relevance of this myristoylation, we created the *Ld*ARL-1/G2A (unMyr) mutant, which cannot be myristoylated, and expressed in *L. amazonensis* promastigotes. As seen on western blots (Fig 2), *Ld*ARL-1/G2A-GFP was expressed at a similar level as native *Ld*ARL-1-GFP and the expression of the endogenous native protein was unmodified. The *in vitro* growth of untagged *Ld*ARL-1/G2A transfectants was comparable to the wild type cells (Fig S2). Remarkably, fluorescence microcopy analyses showed that, unlike the native *Ld*ARL-1-GFP, *Ld*ARL-1/G2A-GFP remained diffuse within the cytoplasm, including the flagellum, within 100% of the cells (Fig 4B). Thus, like in other organisms, *Ld*ARL-1 must be N-myristoylated for its correct targeting to the TGN.

### *Ld*ARL-1 GDP/GTP cycling

G proteins cycle between GDP- and GTP-bound forms. Functional studies of these proteins have been done using mutants representing «GDP-bound» blocked (GDP to GTP exchange deficient) or «GTP-bound» blocked (GTPase-deficient) forms. The most recent characterisation of such mutants concerns human *Hs*ARF-6 [44]: the Q67L mutant represents the GTP-bound blocked form, T44N the GDP-bound blocked form, and the T27N, an empty form devoid of nucleotide, earlier erroneously considered as a GDP-bound form. The empty form of the native protein is a transient intermediate bound to the nucleotide-exchange factor; since the T27N mutant cannot bind GTP, the complex is stable, the exchange process blocked and the cycling also when all the available nucleotide-exchange factor becomes complexed.

Untagged proteins or GFP fusions of the three corresponding mutants, *Ld*ARL-1/Q74L (GTP-bound blocked), /T51N (GDP-bound blocked) and /T34N (empty) were created and stably expressed in *L. amazonensis* promastigotes; similar data were obtained for both series.

**Figure 5:**
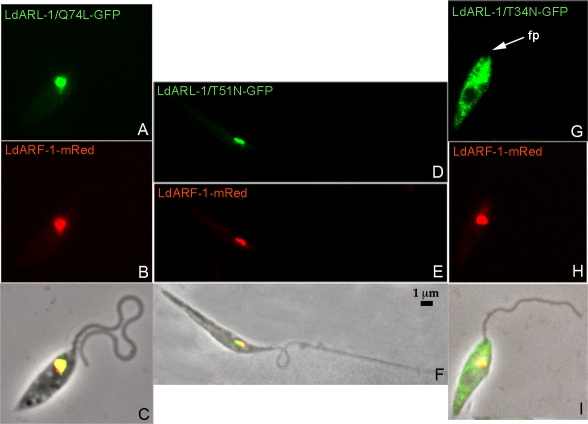
Localisation of *Ld*ARL-1-GFP mutants (GTP-, GDP-bound and empty forms). Fixed *L. amazonensis* promastigotes expressing in red *Ld*ARF-1-mRed (5B, 5E, 5H) and in green *Ld*ARL-1/Q74L-GFP (GTP) (5A), *Ld*ARL-1/T51N-GFP (GDP) (5D), *Ld*ARL-1/T34N-GFP (empty) (5H); overlays are in 5C, 5F and 5I. fp, flagellar pocket.

The growth rate of the cell lines expressing *Ld*ARL-1/Q74L or *Ld*ARL-1/T51N were comparable (Fig S2). Both proteins localised to the TGN (Fig 5A and D) and colocalised with the TGN marker *Ld*ARF-1-mRed (Fig 5B/5C and 5E/5F, respectively). Remarkably, while expressed at similar levels as *Ld*ARL-1/Q74L-GFP (Fig 2), the double-mutant *Ld*ARL-1/G2AQ74L-GFP remained cytoplasmic (not shown), as did *Ld*ARL-1/G2A-GFP, thus confirming that myristoylation is mandatory for the TGN localisation.

The expression of *Ld*ARL-1/T34N-GFP provided different observations. First, despite the comparable amount of endogenous *La*ARL-1 between the different transgenic cell lines, *Ld*ARL-1/T34N-GFP expression level was much lower compared to the other variants (Fig 2). Accordingly, fluorescence microscopy observations revealed that, unlike the other mutants, only 15-20% of the cells were expressing detectable levels of *Ld*ARL-1/T34N-GFP, suggesting that the protein was somehow toxic, and its expression repressed or counterselected. However, the growth rate of this cell line was comparable to the wild type cells (Fig S2). Second, the fluorescence labelling obtained in the *Ld*ARL-1/T34N-GFP-expressing cells was non-homogeneously distributed throughout the cytoplasm and totally excluded from the flagellum (Fig 5G), an observation easily discernable from the one made previously with the G2A mutant (Fig 4B). This is consistent with the TGN disorganisation observed in mammals with the equivalent ARL-1/T31N mutant overexpression [18]. However, the unchanged localisation of *Ld*ARF-1-mRed (Fig 5H) suggests differences in TGN targeting/organisation. Finally, localization of the double-mutant *Ld*ARL-1/G2AT34N (not shown) was undistinguishable from the G2A mutant, confirming again the necessity of N-terminal myristoylation.

In summary, *Ld*ARL-1 entire GDP/GTP cycling occurs within the TGN; the empty form is cytoplasmic.

### *Ld*ARL-1 and intracellular trafficking

The various *Ld*ARL-1 mutants were used to further explore *Ld*ARL-1 function. As the TGN is a complex structure specialised in the sorting of molecules to the plasma membrane, the lysosome and other organelles, the putative involvement of *Ld*ARL-1 in intracellular traffic was investigated.

#### a/Exocytosis and endocytosis

**Figure 6:**
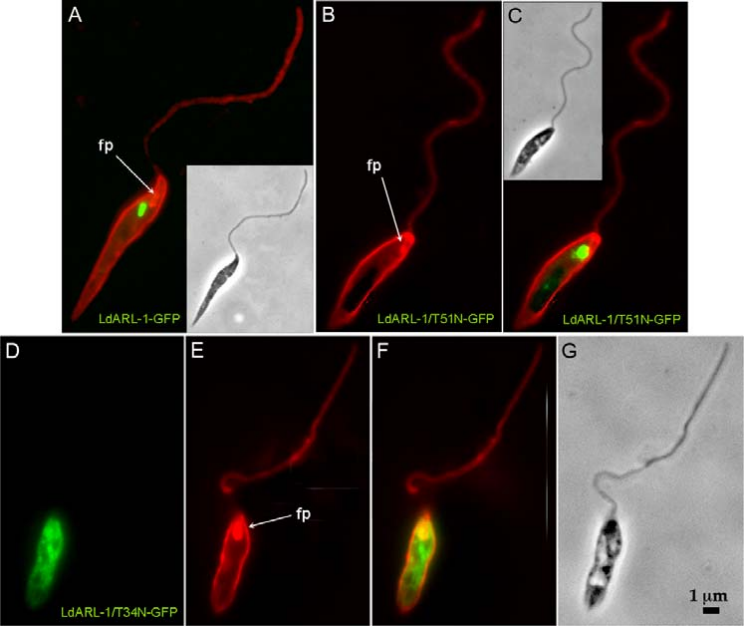
* Ld*ARL-1 and the flagellar pocket. Fixed *L. amazonensis* promastigotes with plasma membrane labelled in red with Concanavalin A-Texas Red. A, cell expressing in green *Ld*ARL-1-GFP. B-C, cell expressing in green *Ld*ARL-1/T51N-GFP (GDP); D-F, cell expressing in green *Ld*ARL-1/T34N-GFP (empty). The yellow signal is an overlay of green and red signals.

The trypanosomatids are polarised cells in which endo- and exocytosis occur solely in a peculiar structure, the flagellar pocket. Upon exogenous expression of *Ld*ARL-1/T34N-GFP (empty), the flagellar pocket of the transgenic *L. amazonensis* cells appeared inflated, forming a gap at the anterior part of the cells (Fig 5G, fp); this was better resolved by labelling the pellicular and pocket membranes with Texas-Red-Concanavalin A, as seen for the cell pictured on Fig 6D–G and comparing to a cell expressing native *Ld*ARL-1-GFP (Fig 6A) or *Ld*ARL-1/T51N-GFP (GDP) (Fig 6B–C). This flagellar pocket inflation was similar to the observation made when endocytosis was inhibited by RNAi ablation of clathrin heavy chain in *T. brucei* [45], but could also result from enhanced exocytosis.

The activity of the secreted acid phosphatase (SAP) was determined in the culture medium of untransformed cells and expressing native or mutated *Ld*ARL-1-GFP; no significant difference was found (Fig S2), suggesting that SAP exocytosis was unmodified.

**Figure 7:**
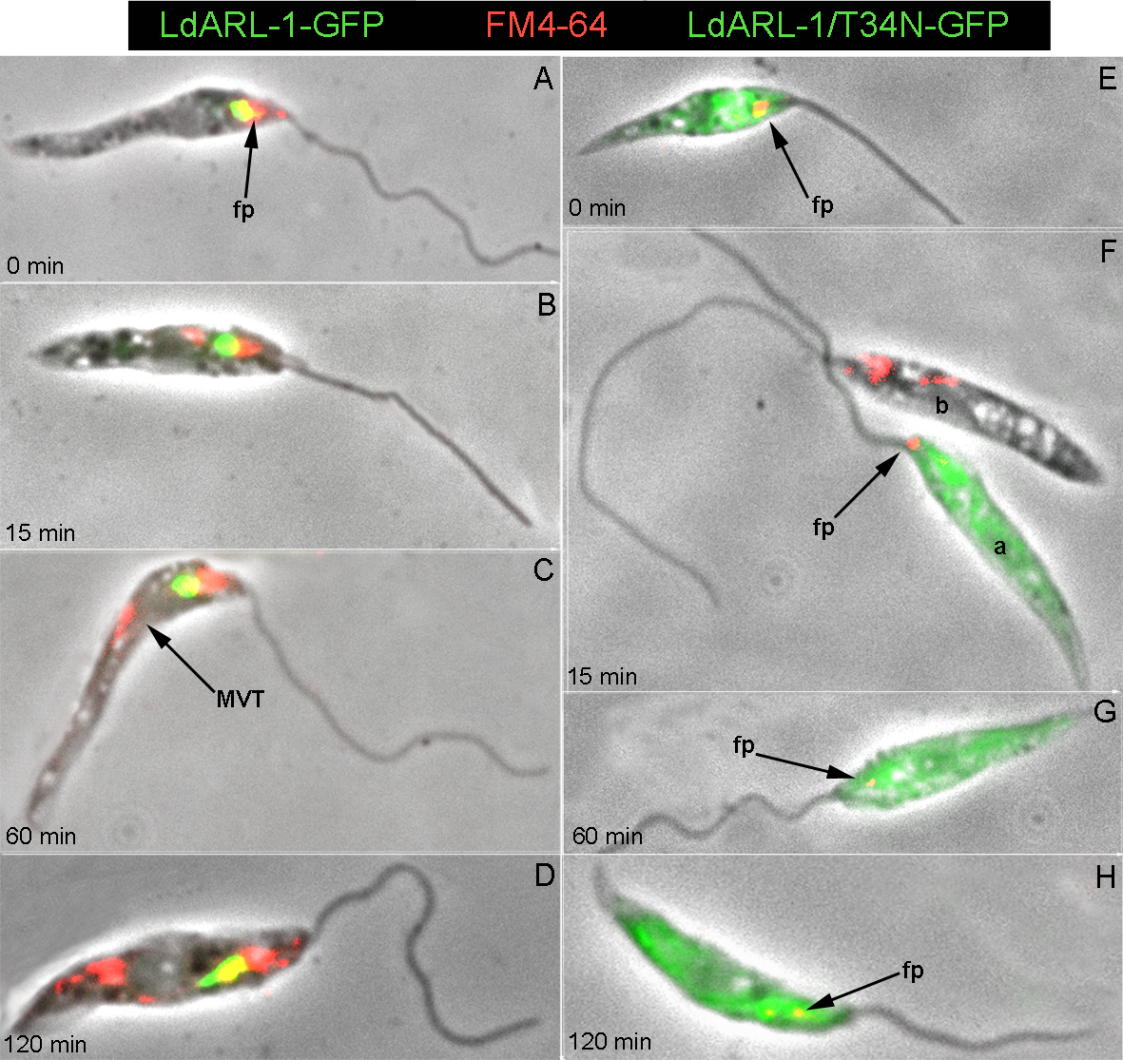
* Ld*ARL-1 and FM4-64 internalisation. *L. amazonensis* promastigotes expressing *Ld*ARL-1-GFP (A–D, green) or *Ld*ARL-1/T34N-GFP (empty) (E–H, green) were incubated at 24°C for 15 min with 2 µg/ml FM4-64FX (red), washed and further incubated for 0 min (A, E), 15 min (B, F), 60 min (C, G) and 120 min (D, H) before fixation; fp, flagellar pocket; MVT, Lysosome/Multivesicular Tubule. Colocalization of red and green results in a yellow signal.

Endocytosis was assessed by FM4-64 pulse-chase experiments (Fig 7). In *Leishmania*, this dye is readily internalised and targeted through endosomes to the final digestive compartment termed Lysosome/Multivesicular Tubule (MVT) [46]. In cells expressing *Ld*ARL-1-GFP (Fig 7A–D), *Ld*ARL-1/Q74L-GFP (not shown) or *Ld*ARL-1/T51N-GFP (not shown), FM4-64 endocytosis occurred as expected, with a progressive and massive FM4-64 labelling from the flagellar pocket to the MVT from 0 to 120 minutes. On the contrary, cells expressing *Ld*ARL-1/T34N-GFP showed endocytosis defect (Fig 7E–H); the FM4-64 uptake was blocked, the labelling, remaining in the area of the flagellar pocket up to 60 minutes (Fig 7E–G, arrows), was only seen progressing to the MVT at later times (120 min) and in much smaller amounts (Fig 7H, arrow); as comparison, the cell a in Fig 7F, which expresses *Ld*ARL-1/T34N-GFP, and the cell b which does not stresses the difference in FM4-64 internalization. This endocytosis defect was not due to clathrin depletion, as revealed by immunofluorescence with an anti-*Tb*Clathrin Heavy Chain immune serum (not shown).

#### b/Traffic to the lysosome/MVT

**Figure 8:**
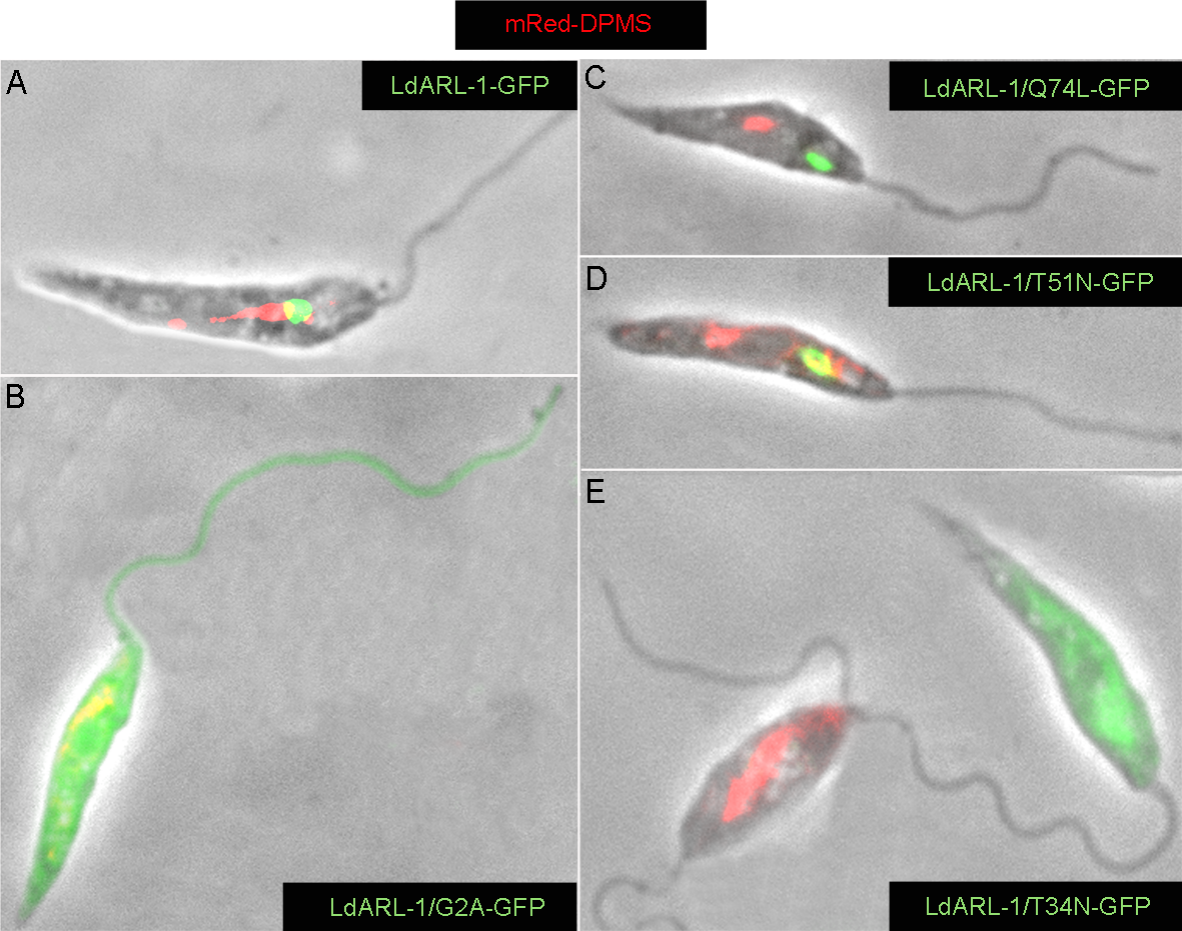
* Ld*ARL-1 and Golgi to Lysosome/MVT traffic. BA125 *L. amazonensis* promastigotes expressing in red the mRed-DPMS and in green *Ld*ARL-1-GFP (A), *Ld*ARL-1/G2A-GFP (unMyr) (B), *Ld*ARL-1/Q74L-GFP (GTP) (C), *Ld*ARL-1/T51N-GFP (GDP) (D) or *Ld*ARL-1/T34N-GFP (empty) (E).

To further investigate the endocytosis deficiency observed in the *Ld*ARL-1/T34N-GFP-expressing cells, the dolichol-phosphate-mannose synthase (DPMS) was used as marker (Fig 8). In *Leishmania*, this membrane-anchored protein enzyme, essential for the biosynthesis of glycophosphatidylinositol anchors of membrane proteins, localises to the transitional Endoplasmic Reticulum [47]. However, GFP-DPMS chimeras are transported, via the Golgi apparatus and multivesicular bodies, to the lysosome/MVT [48]. A similar observation was made with an mRed-DPMS chimera in our cells expressing native *Ld*ARL-1-GFP (Fig 8A), *Ld*ARL-1/G2A-GFP (Fig 8B), *Ld*ARL-1/Q74L-GFP (Fig 8C) and *Ld*ARL-1/T51N-GFP (Fig 8D). In contrast, the mRed-DPMS chimera was undectectable in cells expressing the empty form *Ld*ARL-1/T34N-GFP (Fig 8E); as shown on Fig 8E, there is a clear mutual exclusion between the expression of *Ld*ARL-1/T34N-GFP (cell a) and mRed-DPMS detection (cell b). This suggests an alteration of the secretion pathway and/or a traffic misdirection.

#### c/Traffic to the acidocalcisomes

In trypanosomatids, acidocalcisomes are essential acidic organelles, probably lysosome-related [49]. They contain polyphosphates and calcium. Their acidic pH is maintained via the action of a membrane-bound pump, the proton pyrophosphatase (VP1). In *T. brucei*, *Tb*VP1 is essential for the survival of the mammalian forms of the parasite [50].

**Figure 9:**
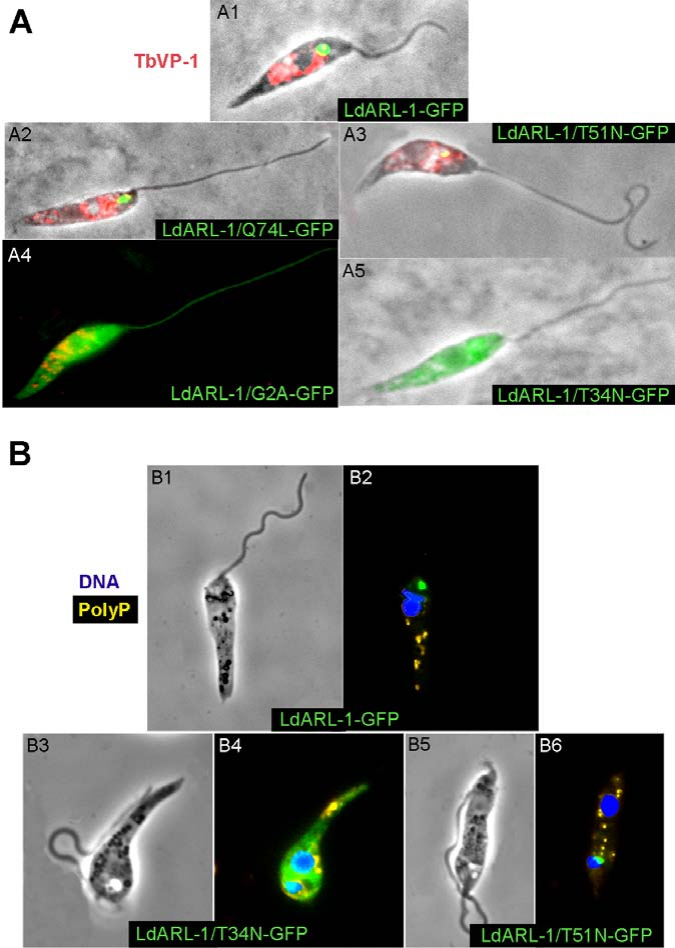
* Ld*ARL-1 and acidocalcisomes. A. Fixed *L. amazonensis* promastigotes expressing in green *Ld*ARL-1-GFP (1), *Ld*ARL-1/Q74L-GFP (GTP) (2), *Ld*ARL-1/T51N-GFP (GDP) (3), *Ld*ARL-1/G2A-GFP (unMyr) (4) or *Ld*ARL-1/T34N-GFP (empty) (5) and stained in red with the rabbit anti-*Tb*VP1 (acidocalcisomes marker) immune serum plus anti-rabbit IgG-Texas-Red conjugate. B. Live *L. amazonensis* promastigotes expressing in green *Ld*ARL-1-GFP (1-2), *Ld*ARL-1/T34N-GFP (empty) (3-4) or *Ld*ARL-1/T51N-GFP (GDP) (5-6) and stained with DAPI: in Blue, nucleus and kinetoplast, in yellow: polyphosphates of acidocalcisomes [50].

The VP1 orthologue exists in *L. amazonensis* (*La*VP1) and can be detected by immunofluorescence, using the anti-*Tb*VP1 immune serum (N. Bakalara, personal communication), within large round-shaped organelles, distributed thoughout the promastigote body. Using the TbVP1 antibody, this characteristic pattern of acidocalcisomes was observed in *L. amazonensis* promastigotes expressing *Ld*ARL-1-GFP (Fig 9A-1), *Ld*ARL-1/Q74L-GFP (Fig 9A-2), *Ld*ARL-1/T51N-GFP (Fig 9A-3) and the delocalised/inert *Ld*ARL-1/G2A-GFP (Fig 9A-4). Strikingly, in cells expressing *Ld*ARL-1/T34N-GFP, *Tb*VP1 was undetectable (Fig 9A-5). However, DAPI staining of live cells, which allows visualisation of their acidocalcisomes by revealing their polyphosphate content, showed that all cells expressing *Ld*ARL-1-GFP (Fig 9B-1), *Ld*ARL-1/T34N-GFP (Fig 9B-2) and *Ld*ARL-1/T51N-GFP (Fig 9B-3) contained polyphosphate/acidocalcisomes; this ruled out the possible absence of these organelles in the *Ld*ARL-1/T34N-GFP expressing cells. Therefore, in *L. amazonensis*, expression of *Ld*ARL-1/T34N-GFP induced a misdirection, possibly exocytosis, of the membrane-bound proton pyrophosphatase.

### *Ld*ARL-1 and GRIP domain proteins

In mammals and yeast, ARL-1/GTP has been shown to bind to the GRIP domain of large coiled-coil proteins, a conserved C-terminal sequence of about 45 residues sufficient to direct these proteins to the TGN [51], [52]. These proteins are vesicle tethering factors [32]. In the Trypanosomatid genomes, only two proteins are predicted to possess GRIP domains. We called them pGRIP-1 (*L. infantum* Linj11.0070 and *L. major* LmjF11.0070 with 1334 amino acids, *T. brucei* Tb11.02.5040 with 613 aa) and pGRIP-2 (*L. infantum* LinJ34.3660 with 488 aa, *L. major* LmjF34.4090 with 435 aa and *T. brucei* Tb927.4.640 with 462 aa). They share homologies solely in the GRIP domains (within trypanosomatids and other species). We isolated the *L. donovani* GRIP domain of pGRIP-1 (called *Ld*GRIP), its *T.brucei* homologue (*Tb*GRIP, which allowed the TGN targeting of the GFP-*Tb*GRIP fusion protein in *L. mexicana* [53]) and for the first time the full-length *Ld*pGRIP-2. The three ORFs were fused with mRed at their N-terminus, leaving the GRIP domain at the C-terminus, and co-expressed in *L. amazonensis* together with *Ld*ARL-1-GFP or its mutants. As the results obtained were similar, only the data obtained with mRed-*Ld*pGRIP-2 will be described.

**Figure 10:**
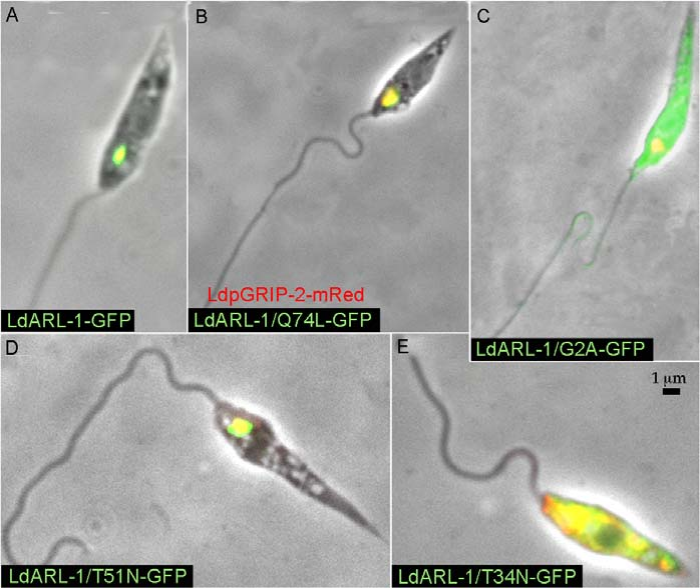
* Ld*ARL-1 and *Ld*pGRIP-2. Overlay of fixed *L. amazonensis* promastigotes expressing in red mRed-*Ld*pGRIP-2 and in green *Ld*ARL-1-GFP (A), *Ld*ARL-1/Q74L-GFP (GTP) (B), *Ld*ARL-1/G2A-GFP (unMyr) (C), *Ld*ARL-1/T51N-GFP (GDP) (D) and *Ld*ARL-1/T34N-GFP (empty) (E). Overlay of red and green is yellow.

Fluorescence microscopy analyses revealed that mRed-*Ld*pGRIP-2 co-localised to the TGN with *Ld*ARL-1-GFP (Fig 10A), *Ld*ARL-1/Q74L-GFP (Fig 10B), and *Ld*ARL-1/T51N-GFP (Fig 10D). Interestingly, *Ld*ARL-1/G2A-GFP, which remained cytoplasmic (cf Fig 4B, 6C), did not interfere with mRed-*Ld*pGRIP-2 localisation (Fig 10C). Even more interestingly, *Ld*ARL-1/T34N-GFP inhibited the TGN addressing of mRed-*Ld*pGRIP-2, as both proteins colocalised within the cytoplasm (Fig 10E). This observation is consistent with the TGN disorganisation observed in mammals by the equivalent mutant ARL-1/T31N overexpression [18] and probably explains the interruption of intracellular traffic from the TGN, since the vesicle tethers are no longer recruited to their location.

## Discussion

### 1. The *Ld*ARL-1 gene in *Leishmania* and other Trypanosomatids

We identified and characterised a trypanosomatid member of the ARF/ARL family, the *Leishmania Ld*ARL-1. The gene was named ARL-1 after the highest percentage of identity with the corresponding mammalian and yeast homologues. It is conserved in other *Leishmania* (*L. infantum*, 100% identity; *L. major*, 3 amino acids are different; *L. amazonensis*, 4 amino acids are different; *L. brasiliensis*, 12 amino acids are different, one is added) and trypanosome species (*T. brucei*, 47 amino acids are different, 3 are added) whose sequence is available.

The situation in *T. cruzi* might be different as two sequences were reported. The first *Tc*ARL-1 has a comparable size to other species (45 different amino acids, 3 added). The second *Tc*ARL-1 has a 101 amino acid N-terminal extension; its ARL-1 domain has 3 different amino acids when compared to the first *Tc*ARL-1; 46 different amino acids, 3 added when compared to *Ld*ARL-1. However, the N-terminal extension is created by the deletion of a T in a stretch of 7 in the vicinity of the 5′ side of the ARL-1 part. There is no obvious polypyrimidine tract upstream of the ATG and the predicted extended *Tc*ARL-1 protein cannot be N-myristoylated because of the absence of a Glycine in position 2. Although there is an example in mammals of a chimeric protein with a C-terminal ARF-1 part and a N-terminal extension playing the role of an internal GAP (GTPase Activating protein) [54], the reality of the *T. cruzi* variant should be investigated in more detail to rule out the possibility of sequencing errors and/or to relate it with the existence of two divergent lineages of *T. cruzi* [55], eventually compatible with a 3 amino acid difference between the two *Tc*ARL-1 regions.

Conservation of the general organisation of the chromosomal region (Fig S1) (even for the extended variant gene of *T. cruzi*) and the comparable percentages of identity between the different neighbouring orthologues across species suggest that all ARL-1s are functional homologues and that the *Tb*ARL-1 gene [15], erroneously annotated as *Tb*ARF-3 (GeneDB http://www.genedb.org/) should be renamed *Tb*ARL-1, unless functional data contradict this assumption.

### 2. N-myristoylation of *Ld*ARL-1

As for other members of the ARF/ARL family, the glycine in position 2 represents a myristoylation site; myristoylation is impossible for the *Ld*ARL-1/G2A-GFP mutated protein which remained diffuse within the cytoplasm, including the flagellum. Still, the mRed-*Ld*pGRIP-2, mRed-*LdGRIP* or mRed*Tb*GRIP localised to the TGN, showing that the *Ld*ARL-1/G2A-GFP was unable to compete with the endogenous native *La*ARL-1 and inhibit its function. Emphasising the essential character of myristoylation, the double-mutants *Ld*ARL-1/G2AQ74L-GFP (unMyr-GTP) and *Ld*ARL-1/G2AT34N-GFP (unMyr-empty) remained cytoplasmic, and their expression had no effect on the GRIP domain protein targeting. The absence of N-terminal myristate probably generates functionally inert *Ld*ARL-1 proteins, unable to bind to membranes and be recognised by a putative membrane bound targeting receptor/mechanism as suggested for *Hs*ARF-6 [56].

### 3. The *Ld*ARL-1 mutants and GDP/GTP cycling

Based on ras protein properties, mutations of conserved amino acids have been created to mimick GDP-bound or GTP-bound forms of the proteins of this superfamily. Thus, the last Threonine of the first motif of the GTP binding site (Fig 1) has been considered essential for GTP binding, its mutation leading to a drastic decrease in GTP affinity, hence producing a GDP-bound blocked form. However, due to the difficulties of determining the affinities for nucleotides, especially when they are low and if the proteins are insoluble, these affinities have been rarely looked over. After the crystal structure of several GDP- or GTP-bound ARF/ARLs has been solved, the validity of the prediction for all the ras family proteins became questionable. In a recent detailed study of human ARF-6 [44], it was clearly shown that the mutant ARF-6/T27N, considered as a GDP-bound blocked form, was in reality an empty form devoid of nucleotide since the Threonine 27 interacted with both GDP and GTP. Accordingly, in our work, the mutant *LdLd*ARL-1/T34N was considered as an empty form, and the new mutant *Ld*ARL-1/T51N, a GDP-blocked form (cf Fig S3).

When expressed in *Leishmania* promastigotes, *Ld*ARL-1/T34N-GFP was delocalised to the cytoplasm, similarly to the equivalent mutants in mammalian cells [18]. However, *Ld*ARF-1-mRed localisation did not change, so that the TGN was not disorganised to the same extent as in mammals [18]. accordingly, it has been shown in mammals that Brefeldin A-induced disorganisation of the Golgi, which includes ARF-1 delocalisation to the cytoplasm, occurs within minutes of drug exposure, while it takes much longer for ARL-1 [17]. We found no effect of Brefeldin A in *Leishmania* (unpublished data), possibly because it was not internalised. Conversely, *Ld*ARL-1/T51N-GFP (GDP) localised to the TGN, and co-localised with *Ld*ARF-1-mRed. It was the same for *Ld*ARL-1/Q74L-GFP (GTP), which was different from mammals, where ARL-1/Q71L (GTP) led to an expansion of the Golgi and a delocalisation of ARF-1 [18]. It then appears that, in *Leishmania*, the entire *Ld*ARL-1 GDP/GTP cycling occurs within the TGN and the delocalisation to the cytoplasm of the «empty form» is a non-physiological event due to the expression of a transient form normally associated to the nucleotide exchange factor; the blocked empty form might act as a sink for the nucleotide exchange factor, thus forming a stable and inactive complex, as has been suggested for the equivalent mutant ARF-1/T31N [57].

From our data, one can predict that the unknown exchange factor is limiting, since the mutant *Ld*ARL-1/T34N-GFP is dominant-negative, i.e. blocks the endogenous native *La*ARL-1 function. The lack of physiological effect of *Ld*ARL-1/T51N-GFP suggests that this form does not stably associate with the exchange factor, the interaction being stabilised by the release of GDP. On the contrary, the unknown GAP is either non-limiting or not blocked by *Ld*ARL-1/Q74L-GFP since expression of this mutant does not block the endogenous native ARL-1 function. There is no obvious Ysl2p homologue in the *Leishmania* genome, the suggested yeast Arl-1p nucleotide exchange factor [24], nor Gcs1p, the suggested Arl-1p GTPase-activating protein [25]; their identification might help to better understand *Ld*ARL-1 GDP/GTP cycling.

These data illustrate the diversity of ARL proteins modes of GDP/GTP cycling. If one compares *Ld*ARL-1 and *Ld*ARL-3A [58], [59], *Ld*ARL-1/GDP- or /GTP-blocked expression does not impair the endogenous protein function, while *Ld*ARL-3A/GDP- or /GTP-blocked expression blocks the endogenous *La*/*Ld*ARL-3A function, resulting in a drastic flagellar shortening; on the other hand, *Ld*ARL-1/T34N (empty) is dominant-negative whereas *Ld*ARL-3A/T30N (empty) is inert. The most likely explanation for these differences lies in the necessity (*Ld*ARL-1) or not (*Ld*ARL-3A) of a nucleotide exchange factor for the GDP/GTP cycle completion.

### 4. *Ld*ARL-1 role in intracellular traffic as revealed by the dominant-negative mutant *Ld*ARL-1/T34N

Expression of *Ld*ARL-1/T34N-GFP significantly perturbed *Leishmania* intracellular trafficking. Endocytosis was completely blocked, trafficking of DPMS from the Golgi to the Lysosome/MVT, and of the membrane-bound vacuolar proton pyrophosphatase to the acidocalcisomes, was interrupted. The fate of these markers remains enigmatic; they were possibly degraded or misdirected and exocytosed, which would be reminiscent of *Sc*Arl-1 KO, where lucifer yellow uptake was reduced and the vacuolar Carboxypeptidase Y secreted instead of being vacuolar [21]. In *Leishmania*, exocytosis seemed unaffected; however, only the SAP marker (secreted acid phosphatase) could be investigated and changes changes in exocytosis might not have been detected since only 15–20% of the cells expressed the mutated protein.

The acidocalcisomal defect observed with *Ld*ARL-1/T34N-GFP expression resembled the vacuolar defect obtained in yeast with the mutant Arl-1p/D151G mutant, which proved also dominant-negative [20], but the mechanism of action is probably different, because, from the protein structures, the D151 of Arl-1p does not interact with GDP or GTP. At least some of the membrane-bound proteins of acidocalcisomes originate from the TGN and a functional *Ld*ARL-1 protein is essential for their transport to their final compartment. Whether *Ld*ARL-1-mediated targeting to acidocalcisomes is direct from the TGN or necessitates endosomal/lysosomal intermediates remains unknown [49] and might be investigated in the future if/when appropriate *Leishmania* protein markers are identified.

### 5. ARL-1-dependent Golgi targeting of GRIP domain containing Golgins

The GRIP domain is a C-terminal 45 aa long domain present in at least five human and one yeast proteins [32], [60]. Essential for the TGN targeting of these proteins, it is conserved in trypanosomatids and in *Leishmania*, a *T. brucei* GRIP domain is sufficient to target GFP to the TGN [53].

Based on two-hybrid and affinity chromatography experiments, it has been shown that the GTP-bound form of *Sc*arl-1p (or *Hs*ARL-1) binds GRIP domains [27], [29], [31]. In the current model (described in yeast but applicable to mammals), the GDP-bound form of *Sc*arl-1p (*Hs*ARL-1) is cytoplasmic; when GDP is exchanged with GTP under the action of the nucleotide exchange factor, Scarl-1p (*Hs*ARL-1) moves to the Golgi membranes and recruits GRIP domain proteins [30] [61], [62]. In this model, it has been considered that the GDP form of *Sc*arl-1p (*Hs*ARL-1) is cytoplasmic, because the mutants Scarl-1p/T32N (*Hs*ARL-1/T31N) mutants are cytoplasmic. It has also been assumed that the GDP-loaded native Scarl-1p (*Hs*ARL-1) is equivalent to the Scarl-1p/T32N (*Hs*ARL-1/T31N) mutant, which is probably inaccurate if these mutants are «empty forms».

In *Leishmania*, there are two proteins with GRIP domains, which we called *Ld*pGRIP-1 and *Ld*pGRIP-2. *Ld*pGRIP-2 and the GRIP domains of *Ld*pGRIP-1 (*Ld*GRIP) and *Tb*pGRIP-1 (*Tb*GRIP) were fused to the C-terminus of the mRed protein: mRed-*Ld*pGRIP-2 and mRed-*Ld*GRIP/-*Tb*GRIP co-localised with all *Ld*ARL-1 mutants, except the inert non-myristoylated one. More specifically, mRed-*Ld*pGRIP-2 and mRed-*Ld*GRIP/-*Tb*GRIP were de-localised to the cytoplasm with *Ld*ARL-1/T34N-GFP. From our data, *Ld*ARL-1/T51N-GFP is TGN-specific, while *Ld*ARL-1/T34N-GFP is cytoplasmic. Since the «empty form» and GRIP domain proteins probably do not interact physically, these cytoplasmic localisations probably represent a common consequence of the TGN destabilisation, whixh may not be complete since ARF-1-mRed localisation did not change (within the limits of the fluorescence micoscopy resolution).

At least for *Leishmania*, the model of ARL-1/GRIP domain interaction and Golgi recruitment might be slightly modified. GRIP domain proteins and ARL-1 under both GDP and GTP forms are localised to the TGN. Both *Ld*ARL-1 forms are probably associated to membranes as *Hs*ARF-6 [56]. The GTP form may recruit GRIP-domain proteins from the Golgi soluble compartment to Golgi membranes and the GDP form releases them. This is consistent with the fact that in Brefeldin A-treated mammalian cells, ARL-1 is delocalised from the Golgi membranes while GRIP domain proteins are not [27], showing that, at least in mammals, ARL-1 is not necessary for the maintenance of GRIP domain proteins in the TGN. In the case of Arl1p knockout in yeast, the GRIP domain protein Imh1p was mislocalised [29] possibly because it could not reach the Golgi at all.

The recruitment of GRIP domain proteins to the Golgi also requires the presence of another small G protein, Scarl3p, and an integral membrane protein, ScSys1p [61], [62]; Scarl3p/GTP and /GDP forms were included in the study, but the mutant used for the GDP form might also be an «empty form»; the model should be revisited according to this new interpretation.

Concerning *Leishmania*, we have found a putative *Sc*arl-3p homologue (not yet functionnaly investigated), but there is no obvious candidate for *Sc*Sys1p. Much work remains to be done for the understanding of this aspect of the Golgi structure/function.

### 6. Significance of *Ld*ARL-1 in the biology of *Leishmania* cells

The *Ld*ARL-1 gene is expressed in both promastigotes (insect forms) and amastigotes (mammalian forms) of *L. donovani* and *L. amazonensis*. This differs from *T. brucei*, where *Tb*ARL-1 is expressed only in the bloodstream forms of the parasite (i.e. the mammalian forms) and is essential for their viability, as revealed by RNAi experiments [15]. Although it has not been formally demonstrated and in spite of being annotated as ARF-3 in the *T. brucei* database (Tb927.7.6230, GeneDB http://www.genedb.org/), this *Tb*ARL-1 is very likely the functional homologue/orthologue of *Ld*ARL-1 given the synteny of the homologous chromosomal region of several trypanosomatids (Fig S1). The differential expression seen in *T . brucei* forms probably reflects differences in the physiology of these cells: endocytosis is minimal in *T. brucei* insect forms, while it is extraordinarily active in bloodstream forms [4], which correlates well with *Ld*ARL-1 involvement in endocytosis. In *Leishmania*, *Ld*ARL-1 and endocytosis must be active in all forms of the parasite.

Finally, the fact that *Ld*ARL-1 could not complement the yeast arl-1Δ deletion mutant [21] (not shown) reveals that, even for evolutionary conserved pathways such as intracellular traffic, inter-species differences exist, which might hopefully be exploited against deleterious (from the human point of view only) organisms in the future.

## Materials and Methods

### Parasites, animals and immune sera

Promastigotes of *L. donovani* (MHOM/SD/00/Khartoum: LSB51.1) and *L. amazonensis* (MHOM/BR/1987/BA125; MPRO/BR/1972/M1841 = LV79) were cultured at 24°C in AM medium [58], [63]. Parental and transformed *L. amazonensis* promastigotes growth was monitored everyday after seeding at a density of 2,5×10^6^/ml by counting with a Malassez hemocytometer. Balb/c mice (Centre d'Elevage Janvier) were infected in footpads with 5 10^6^ *L. amazonensis* promastigotes; amastigotes were recovered from lesions as previously described [58].

An anti-*Ld*ARL-1 C-terminus rabbit immune serum was prepared as described for *Ld*ARL-3A [58] using an *Ld*ARL-1 C-terminal synthetic peptide (NH_2_-MDWLVERLREQGIGA-COOH, Genosphere Biotech) and a «Fauve de Bourgogne» rabbit (Centre d'Elevage Janvier). The rabbit anti-T*b*VP1 immune serum has been described [50], [64]. The rabbit anti-LACK immune serum [65] was kindly provided by Dr J.-C. Antoine and the rabbit anti-*Tb*Clathrin heavy chain immune serum [66] by Dr M. Field.

Protein extraction and western blotting were done as previously described [58] except for the revelation, done with a rabbit anti IgG peroxidase conjugate (1∶10000 dilution) and an ECL revelation kit (Amersham). The secreted acid phosphatase (SAP) activity was determined as previously [58] according to [67]. Briefly, after seeding the cells at a density of 2.5 10^6^ per ml, aliquots of the culture medium were removed every following day for 5 days, filtered through a 0.22 µm pores membrane to remove cells and debris; 138 µl supernatant were then incubated in a final volume of 200 µl with 50 mM sodium acetate pH 4, 0.1% β-mercaptoethanol (v/v), 50 mM para-nitrophenyl phosphate for 30 min at 37°C; the reaction was stopped with 800 µl sodium hydroxide 2 M and the absorbance of the released para-nitrophenolate at 410 nm determined; the amount of para-nitrophenolate ion was calculated considering an extinction coefficient of 17.8 mM^−1^, and the data presented as nmoles para-nitrophenyl phosphate hydrolyzed per minute and milliliter of supernatant.

### Isolation of the *Ld*ARL-1 gene

144 bp PCR fragments were amplified with oligonucleotides G019 (5′ GGIYTIGAYDVIGCIGCIAARAC 3′)/G020 (5′ YTGICCICCIAIRTCCCA 3′) from *L. donovani* genomic DNA, cloned into the pUC18 vector and sequenced. The 4020 fragment was used as a probe to screen a set of filters of the *L. donovani* LSB-51.1 genomic cosmid Stuartlab-Cos library (gift of Drs T. DeVos, P. Myler and K. Stuart). The complete *Ld*ARL-1 ORF was sequenced on cosmid n°7227, recovered with primers G039 (5′ tgtggatcccatATGGGTGCGTGGCTGAC 3′) /G043B (5′ tgt ggatcc GGTTGGCCAGGCGGTCA 3′) and cloned into the *Bam*HI site of pBluescriptSK- (pORF-*Ld*ARL-1).

### Construction of plasmids

For protein expression in *L. amazonensis*, two types of vectors were used. The pTEX vector [38] confers G-418 resistance and allows high protein expression. The pNUS series [39] allows moderate expression of proteins fused either to the Green fluorescent protein (GFP) at their C-terminus (pNUS-GFPcH, conferring hygromycin resistance) or to the monomeric Red fluorescent protein (mRFP [68]) at their N/C terminus (pNUS-mREDnD/-cD conferring blasticidin resistance). All constructs were sequenced prior *Leishmania* transfection.

The *Ld*ARL-1 ORF was recovered from pORF-*Ld*ARL-1 by *BamH*I digestion and cloned into the pTEX vector at the *BamH*I site (pTEX-*Ld*ARL-1) and the pNUS-GFPcH vector between *Nde*I and *Kpn*I after PCR-amplification from pORF-*Ld*ARL-1 with primers G039/G110 (5′ tgtggtaccAGCACCGATCCCTTGCT 3′) to generate pNUS-*Ld*ARL-1-GFPcH. The *Ld*ARL-1/G2A mutant was created by PCR amplification from pORF-*Ld*ARL-1 with the Mut5B (5′ tgtggatcccatATGG**C**TGCGTGGCTGACA 3′; **C** represents the mutated nucleotide)/G043B primers, and cloned into the pTEX and the pNUS-GFPcH as above, generating pTEX-*Ld*ARL-1/G2A and pNUS-*Ld*ARL-1/G2A-GFPcH. The *Ld*ARL-1/T34N mutant was created by site-directed mutagenesis of pORF-*Ld*ARL-1, using oligonucleotides Mut6 (5′ CCGATAGAGAATTGAA**T**TCTTTCCCGC 3′; **T** represents the mutated nucleotide)/Mut8 (5′ CAAAAGCTGGAGC**C**CCACCGCGGTGG 3′; **C** inactivates the vector *Sac*I site) and the Transformer Site-Directed Mutagenesis Kit (Clontech) according to manufacturer's instructions, generating pORF-*Ld*ARL-1-T34N; the ORF was subsequently recloned into the pTEX and the pNUS-GFPcH as above (pTEX-*Ld*ARL-1/T34N and pNUS-*Ld*ARL-1/T34N-GFPcH). The double-mutant *Ld*ARL-1/G2AT34N was obtained by amplification from *Ld*ARL-1-T34N with primers Mut5B/G110 and cloned into the pNUS-GFPcH (pNUS-*Ld*ARL-1/G2AT34N-GFPcH) as above. The *Ld*ARL-1/Q74L mutant was created by nested PCR amplification from pORF-*Ld*ARL-1, using first oligonucleotides G039/Q20B (5′ TCGCACACCCGTT**A**GGCCGCCAAGGTCCC 3′; **A** represents the mutated nucleotide) and Q20A (reverse complement of Q20B)/G043B, then G039/G043B with a mixture of both previously amplified fragments; the mutated ORF was then recloned into pTEX and pNUS-GFPcH as above (pTEX-*Ld*ARL-1/Q74L and pNUS-*Ld*ARL-1/Q74L-GFPcH). The double-mutant *Ld*ARL-1/G2AQ74L was obtained by amplification from *Ld*ARL-1-Q74L with primers Mut5B/G040B (5′ acaggatccTCACTAAGCACCGATCCCTTG 3′) and cloned into the pNUS-GFPcH (pNUS-*Ld*ARL-1/G2AQ74L-GFPcH) as above. The *Ld*ARL-1/T51N mutant was created by nested PCR amplification from pORF-*Ld*ARL-1, using first oligonucleotides G039/G287 (5′ ACTCCCACA**T**TGGGGACGGTG 3′; **T** represents the mutated nucleotide) and G286 (reverse complement of G287)/G110, then G039/G110 with a mixture of both previously amplified fragments; the mutated ORF was then recloned into the pNUS-GFPcH as above (pNUS-*Ld*ARL-1/T51N-GFPcH) and into the pTEX at the *Bam*HI site after PCR amplification from pNUS-*Ld*ARL-1/T51N-GFPcH with G039/G040B (5′ acaggatccTCACTAAGCACCGATCCCTTG 3′)(pTEX-*Ld*ARL-1/T51N). The *La*ARL-1 sequence has been dertermined after PCR amplification from *L. amazonensis* genomic DNA with primers G222 (5′ tatgcgcccggg**ATG**GGTGCGTGGCTGA 3′)/G328 (5′ acaaagcttctcgag**TCACTA**AGCACCGATCCCTTG 3′).

The *Ld*LPG-2 ORF was amplified from *L. donovani* LV9 genomic DNA using oligonucleotides G162 (5′ actcagatctcatATGAACCATACTCGCTCTGTG 3′)/G163 (5′ atctggtaccCTACTCAGATTTGGAAGTGTC 3′), as designed from the *Ld*LPG-2 sequence (Genbank U26175) and cloned between the *Bgl*II and *Kpn*I sites of pNUS-mREDnD (pNUS-mREDnD-*Ld*LPG-2).

The *Ld*ARF-1 ORF was amplified from *L. donovani* LV-9 genomic DNA with oligonucleotides G170 (5′ gatccat**ATG**GGACAGTGGTTGGCCTCC 3′)/G171 (5′ gatcggtaccTTGCATGGTCGCAGAGATGTTG 3′), as designed from the *Ld*ARF-1 sequence (Genbank AY157971) and cloned into the vector pNUS-mRedcD between *Nde*I and *Kpn*I (pNUS-*Ld*ARF-1-mRednD).

The *Tb*GRIP domain (nt 1284–1842, Genbank BK000167 = Tb11.02.5040, GeneDB http://www.genedb.org/) [53] was PCR-amplified from *T. brucei* TREU 927 genomic DNA with primers G213 (5′ gatcagatctAGCTCTTTAGTTTCGCCCGATG 3′)/G214 (5′ gatcggtaccTTACTTCAATGGGGGACACTG 3′); the *Bgl*II/*Kpn*I restricted product was subsequently inserted into the pNUS-mREDnD digested with the same enzymes (pNUS-mREDnD-*Tb*GRIP). The homologous region (*Ld*GRIP) of the *L. donovani* orthologue (*Ld*pGRIP-1) was PCR-amplified from *L. donovani* LV9 genomic DNA with primers G273 (5′ gatccatATGTTGAAGCGTGCATCGACTCAAG 3′)/G274 (5′ gatcggtaccGTGAAATCGAGGGCATCG 3′), as designed from nt 3421-4002 of LinJ11.0070 (GeneDB http://www.genedb.org/), and cloned between the *Nde*I and *Kpn*I sites of pNUS-EYFPcD (pNUS-EYFPcD-*Ld*GRIP). The complete ORF of the *L. donovani* LV9 orthologue of *L. infantum* LinJ34.3660 (GeneDB http://www.genedb.org/), *Ld*pGRIP-2, was PCR-amplified with G290 (5′ ttcagatctcatATGAGCTCTACGGCTAATCG 3′)/G291 (5′ ttcagatctTCATTGCAGCATAGCCTTGG 3′) and cloned into the *Bgl*II site of pNUS-mREDnD (pNUS-mREDnD-*Ld*pGRIP-2).

The *L. mexicana* Dolichol-Phosphate-Mannose Synthase (DPMS) [69] was PCR-amplified from its cDNA (kind gift of Dr M. McConville) with primers G293 (5′ ctcagatctcatATGCAGTACTCCATTATCGTT 3′)/G294 (5′ ctcggtaccCTAGAAGAGGGAATGGTAGAG 3′) and cloned between the *Bgl*II and *Kpn*I sites of pNUS-mREDnD (pNUS-mREDnD-*Lmx*DPMS).

### *L. amazonensis* transformation and fluorescence microscopy

*L. amazonensis* promastigotes were electroporated and transformants selected as described [58] with 50 µg/ml G-418 (Life Technologies), 50 µg/ml hygromycin (Euromedex) or 10 µg/ml blasticidin (InvivoGen). At least two independent electroporations were done for each construct.

Immunofluorescence was done as described [58]. Briefly, cells were spread onto poly-L-Lysine-treated coverslips and fixed for 1 hour with 2% paraformaldehyde at 24°C. The antisera used were the rabbit anti-*Ld*ARL-1 C-terminus (1/1000 dilution), the rabbit anti-T*b*VP1 (1/1000 dilution) and the rabbit anti-*Tb*Clathrin heavy chain (1/100 dilution). For the latter, an additional cell permeabilisation step (10 min with 0,1% Triton X-100) was done after fixation. Secondary antibodies (Molecular Probes) were FITC-conjugated goat anti-rabbit IgG (10 µg/ml) for *Ld*ARL-1 or Texas Red-conjugated goat anti-rabbit IgG (20 µg/ml) for *Tb*VP-1 and *Tb*Clathrin. DNA was stained by adding 10 µg/ml DAPI in the last 5 min incubation with the secondary antibody. Coverslips were washed and mounted on microcope slides with Mowiol [58]. For GFP or mRed fusion proteins, the cells were washed immediately after paraformaldehyde fixation and mounted.

For endocytosis monitoring, cells (10^7^/ml) were incubated in culture medium with 10% FCS and 2 µg/ml FM4-64FX (Molecular Probes) for 15 min at 24°C in the dark, harvested, resuspended in fresh medium, and incubated for various times at 24°C. After two cold PBS washes, cells were spread onto coverslips, fixed 15 min at 4°C with 4% paraformaldehyde, washed and mounted.

For the flagellar pocket and plasma membrane visualisation, cells were washed twice with RPMI-1640 medium plus 1% goat serum and incubated in RPMI-1640 plus 50 µg/ml biotin-labelled Concanavalin A (Sigma) for 30 min at 24°C. After two PBS washes, cells were fixed for 1 hour at room temperature with 2% paraformaldehyde and further incubated for 1 hour with 10 µg/ml Texas-Red-Streptavidine conjugate (Molecular Probes).

For acidocalcisomal pyrophosphate staining [50], cells were washed twice in 116 mM NaCl, 5 mM KCl, 0.8 mM MgSO4, 5.5 mM glucose, 50 mM K-Hepes, pH 7.4, and incubated for 10 min at 30°C in PBS with 10 µg/ml DAPI. Cells were spread on coverslips by centrifugation and observed alive quickly thereafter.

Observations were done with an Axioplan 2 Zeiss fluorescence microscope and a 100× oil lens. Images were acquired with a Princeton Instruments camera and analysed with Metaview™ (Universal Imaging).

### Electron microscopy

Electron microscopy was done as described [70] with slight modifications. 40 ml of exponentially growing pNUS-*Ld*ARL-1-GFP-transformed *L. amazonensis* promastigotes were collected by centrifugation, washed in PBS, fixed, dehydrated and embedded in LR Gold resin. Ultra-thin sections were cut, neutralised in glycine, blocked and incubated in anti-*Ld*ARL-1 rabbit serum diluted 1:5 in 0.5% Tween-20, 0.1% BSA in PBS for 2 h. Sections were washed, incubated in anti-rabbit IgG-10 nm gold 1:30 in 0.5% Tween-20, 0.1% BSA in PBS and processed as described [70].

## Supporting Information

Figure S1 The ARL-1 chromosomal region in trypanosomatids. Genomic map compiled from the Gene DB Parasite Genome database (GeneDB http://www.genedb.org/) [1], [2], [71], [72]. L. major LmARL-1: LmjF17.0070; L. infantum LinfARL-1: LinJ17.0080; L. brasiliensis LbrARL-1: LbrM17_V2.0080; T. brucei TbARL-1: Tb927.7.6230, previously Tb07.2F2.550, annotated as TbARF-3); T. cruzi TcARL-1: two entries: Tc00.1047053506513.60 and Tc00.1047053508919.60. The general organisation is conserved downstream of the ARL-1 ORF, while on the upstream side, several successive insertions and gene duplications occurred in Leishmania (L. brasiliensis>L. major>L. infantum) compared to the trypanosomes.
(13.55 MB TIF)

Figure S2 Growth of L. amazonensis strains and SAP activity in the supernatant. Cells. BA125 parental strain, cyan. Promastigotes transformed with pTEX-LdARL-1, brown; pTEX-LdARL-1/G2A, yellow; pTEX-LdARL-1/Q74L, light green; pTEX-LdARL-1/T34N, blue; pTEX-LdARL-1/T51N, orange; pNUS-LdARL-1-GFP, dark green. Top panel: Growth of parental and transformed L. amazonensis promastigotes. Cells were seeded at a density of 2.5 106 cells/ml and counted for the 5 following days; the mean of 2–5 different experiments is indicated with the standard deviation. The maximal cell density appeared a bit lower for some clones, it is not known if these changes are significant. In fact, the level of trangenes expression cannot be accurately controlled; fluorescence microscopy observations revealed that there are variations from cell to cell, from clone to clone or within the same clone after different periods of culture; a more reliable observation might be made with integrated transgene after homologous recombination but it is not known if the experiment is possible at all. Bottom panel: Secreted acid phosphatase (SAP) activity in the culture supernatant. The enzyme activity was determined as in [58] 2–5 days after seeding, and is expressed in nmoles of PNPP hydrolyzed per min and ml of medium. The complete experiment was done once.
(9.90 MB TIF)

Figure S3 Interactions of Threonines with GTP and GDP. ARF-6 and ARL-1 belong to the same subfamily of ras proteins and their GTP binding sites are well conserved (Fig 1). The structures of two ARL-1 orthologues, the S. cerevisiae ScARL-1/GDP [73] and the human HsARL-1/GTP [74], [75], have been determined. Using the software Swiss-PdbViewer/DeepView for OSX v3.9b1 (http://ca.expasy.org/spdbv/), and the structures of ScARL-1/GDP (Panel A) and HsARL-1/GTP (Panel B), the threonines interacting with the GDP (Panel A) or GTP (Panel B) were selected without modifying their relative positions. Electron density and H-bonds (Green) were emphasised. N = blue, O = red, C = white, H = cyan, P = orange. Similarly to Threonine T27 of HsARF-6, the equivalent threonines T32 of ScARL-1 (Panel A) and T31 of HsARL-1 (Panel B) interact via H-bonds with the α and β phosphorus of GDP (Panel A) and GTP (Panel B). Conversely, like Threonine T44 of HsARF-6, the equivalent Threonine T49 of ScARL-1 (Panel A) does not interact with GDP but the equivalent Threonine T48 of HsARL-1 (Panel B) does interact with GTP. The neighbouring sequences are conserved for all ARL-1 proteins, including LdARL-1 (Fig 1); one might relatively safely predict that the mutant proteins LdARL-1/T34N, ScARL-1/T32N, and HsARL-1/T31N (equivalent to HsARF-6/T27N) lose significantly their affinity not only for GTP but also for GDP, so that these proteins are « empty ». Similarly, the mutations T44N of HsARF-6, T49N of ScARL-1, T48N of HsARL-1 and T51N of LdARL-1 impair the binding of GTP but not GDP, leading to a « GDP-blocked form ».
(6.70 MB TIF)

## References

[1] Ivens AC, Peacock CS, Worthey EA, Murphy L, Aggarwal G (2005). The genome of the kinetoplastid parasite, *Leishmania major*.. Science.

[2] Peacock CS, Seeger K, Harris D, Murphy L, Ruiz JC (2007). Comparative genomic analysis of three *Leishmania* species that cause diverse human disease.. Nat Genet.

[3] Howell GJ, Holloway ZG, Cobbold C, Monaco AP, Ponnambalam S (2006). Cell biology of membrane trafficking in human disease.. Int Rev Cytol.

[4] Overath P, Engstler M (2004). Endocytosis, membrane recycling and sorting of GPI-anchored proteins: *Trypanosoma brucei* as a model system.. Mol Microbiol.

[5] Pasqualato S, Renault L, Cherfils J (2002). Arf, Arl, Arp and Sar proteins: a family of GTP-binding proteins with a structural device for ‘front-back’ communication.. EMBO Rep.

[6] Nie Z, Hirsch DS, Randazzo PA (2003). Arf and its many interactors.. Curr Opin Cell Biol.

[7] Li Y, Kelly WG, Logsdon JM, Schurko AM, Harfe BD (2004). Functional genomic analysis of the ADP-ribosylation factor family of GTPases: phylogeny among diverse eukaryotes and function in *C. elegans*.. FASEB J.

[8] Kahn RA, Cherfils J, Elias M, Lovering RC, Munro S (2006). Nomenclature for the human Arf family of GTP-binding proteins: ARF, ARL, and SAR proteins.. J Cell Biol.

[9] D'Souza-Schorey C, Chavrier P (2006). ARF proteins: roles in membrane traffic and beyond.. Nat Rev Mol Cell Biol.

[10] Porter-Kelley JM, Gerald NJ, Engel JC, Ghedin E, Dwyer DM (2004). *Ld*ARF1 in Trafficking and Structural Maintenance of the trans-Golgi Cisternal Network in the Protozoan Pathogen *Leishmania donovani*.. Traffic.

[11] Price HP, Stark M, Smith B, Smith DF (2007). TbARF1 influences lysosomal function but not endocytosis in procyclic stage *Trypanosoma brucei*.. Mol Biochem Parasitol.

[12] Price HP, Stark M, Smith DF (2007). *Trypanosoma brucei* ARF1 Plays a Central Role in Endocytosis and Golgi-Lysosome Trafficking.. Mol Biol Cell.

[13] Schürmann A, Breiner M, Becker W, Huppertz C, Kainulainene H (1994). Cloning of two novel ADP-ribosylation factor-like proteins and characterization of their differential expression in 3T3-L1 cells.. J Biol Chem.

[14] Hong JX, Lee FJS, Patton WA, Lin CY, Moss J (1998). Phospholipid- and GTP-dependent activation of cholera toxin and phospholipase D by human ADP-ribosylation factor-like protein 1 (HARL1).. J Biol Chem.

[15] Price HP, Panethymitaki C, Goulding D, Smith DF (2005). Functional analysis of *Tb*ARL1, an N-myristoylated Golgi protein essential for viability in bloodstream trypanosomes.. J Cell Sci.

[16] Tamkun JW, Kahn RA, Kissinger M, Brizuela BJ, Rulka C (1991). The ARF-like gene encodes an essential GTP-binding protein in *Drosophila*.. Proc Natl Acad Sci USA.

[17] Lowe SL, Wong SH, Hong WJ (1996). The mammalian ARF-like protein 1 (ArI1) is associated with the Golgi complex.. J Cell Sci.

[18] Lu L, Horstmann H, Ng C, Hong W (2001). Regulation of Golgi structure and function by ARF-like protein 1 (Arl1).. J Cell Sci.

[19] Lee FJS, Huang CF, Yu WL, Buu LM, Lin CY (1997). Characterization of an ADP-ribosylation factor-like 1 protein in *Saccharomyces cerevisiae*.. J Biol Chem.

[20] Abudugupur A, Mitsui K, Yokota S, Tsurugi K (2002). An ARL1 mutation affected autophagic cell death in yeast, causing a defect in central vacuole formation.. Cell Death Differ.

[21] Rosenwald AG, Rhodes MA, Van Valkenburgh H, Palanivel V, Chapman G (2002). ARL1 and membrane traffic in *Saccharomyces cerevisiae*.. Yeast.

[22] Moss J, Vaughan M (1995). Structure and function of ARF proteins: activators of cholera toxin and critical components of intracellular vesicular transport processes.. J Biol Chem.

[23] Vaughan M, Moss J (1997). Activation of toxin ADP-ribosyltransferases by the family of ADP-ribosylation factors.. Adv Exp Med Biol.

[24] Jochum A, Jackson D, Schwarz H, Pipkorn R, Singer-Kruger B (2002). Yeast Ysl2p, homologous to Sec7 domain guanine nucleotide exchange factors, functions in endocytosis and maintenance of vacuole integrity and interacts with the Arf-Like small GTPase Arl1p.. Mol Cell Biol.

[25] Liu YW, Huang CF, Huang KB, Lee FJ (2005). Role for Gcs1p in regulation of Arl1p at trans-Golgi compartments.. Mol Biol Cell.

[26] Ding M, Vitale N, Tsai SC, Adamik R, Moss J (1996). Characterization of a GTPase-activating protein that stimulates GTP hydrolysis by both ADP-ribosylation factor (ARF) and ARF-like proteins - Comparison to the ARD1 GAP-domain.. J Biol Chem.

[27] Van Valkenburgh H, Shern JF, Sharer JD, Zhu X, Kahn RA (2001). ADP-ribosylation factors (ARFs) and ARF-like 1 (ARL1) have both specific and shared effectors: characterizing ARL1-binding proteins.. J Biol Chem.

[28] Setty SRG, Shin ME, Yoshino A, Marks MS, Burd CG (2003). Golgi Recruitment of GRIP Domain Proteins by Arf-like GTPase 1 Is Regulated by Arf-like GTPase 3.. Curr Biol.

[29] Panic B, Whyte JR, Munro S (2003). The ARF-like GTPases Arl1p and Arl3p Act in a Pathway that Interacts with Vesicle-Tethering Factors at the Golgi Apparatus.. Curr Biol.

[30] Jackson CL (2003). Membrane Traffic: Arl GTPases Get a GRIP on the Golgi.. Curr Biol.

[31] Lu L, Hong W (2003). Interaction of Arl1-GTP with GRIP domains recruits autoantigens Golgin-97 and Golgin-245/p230 onto the Golgi.. Mol Biol Cell.

[32] Munro S (2005). The Arf-like GTPase Arl1 and its role in membrane traffic.. Biochem Soc Trans.

[33] Lake JA, Cruz VFDL, Ferreira PCG, Morel C, Simpson L (1988). Evolution of Parasitism: Kinetoplastid Protozoan History Reconstructed from Mitochondrial rRNA Gene Sequences.. Proc Natl Acad Sci USA.

[34] Bringaud F, ller M, Cerqueira GC, Smith M (2007). Members of a Large Retroposon Family Are Determinants of Post-Transcriptional Gene Expression in *Leishmania*.. PLoS Pathogens.

[35] Curotto de Lafaille MA, Laban A, Wirth DF (1992). Gene expression in *Leishmania*: analysis of essential 5′ DNA sequences.. Proc Natl Acad Sci USA.

[36] Kjeldgaard M, Nyborg J, Clark BFC (1996). Protein motifs.10. The GTP binding motif: Variations on a theme.. FASEB J.

[37] Sahin A, Tetaud E, Merlin G, Santarelli X (2005). *Ld*ARL-1 His-tagged recombinant protein: purification by immobilized metal affinity expanded bed adsorption.. J Chromatogr B.

[38] Kelly JM, Ward HM, Miles MA, Kendall G (1992). A shuttle vector which facilitates the expression of transfected genes in *Trypanosoma cruzi* and *Leishmania*.. Nucleic Acids Res.

[39] Tetaud E, Lecuix I, Sheldrake T, Baltz T, Fairlamb AH (2002). A new expression vector for *Crithidia fasciculata* and *Leishmania*.. Mol Biochem Parasitol.

[40] Ma DQ, Russell DG, Beverley SM, Turco SJ (1997). Golgi GDP-mannose uptake requires *Leishmania* LPG2 - A member of a eukaryotic family of putative nucleotide-sugar transporters.. J Biol Chem.

[41] Honda A, Al-Awar OS, Hay JC, Donaldson JG (2005). Targeting of Arf-1 to the early Golgi by membrin, an ER-Golgi SNARE.. J Cell Biol.

[42] Boutin JA (1997). Myristoylation.. Cell Signal.

[43] Price HP, Menon MR, Panethymitaki C, Goulding D, McKean PG (2003). Myristoyl-CoA:protein N-myristoyltransferase, an essential enzyme and potential drug target in kinetoplastid parasites.. J Biol Chem.

[44] Macia E, Luton F, Partisani M, Cherfils J, Chardin P (2004). The GDP-bound form of Arf6 is located at the plasma membrane.. J Cell Sci.

[45] Allen CL, Goulding D, Field MC (2003). Clathrin-mediated endocytosis is essential in *Trypanosoma brucei*.. EMBO J.

[46] Waller RF, McConville MJ (2002). Developmental changes in lysosome morphology and function *Leishmania* parasites.. Int J Parasitol.

[47] Ilgoutz SC, Mullin KA, Southwell BR, McConville MJ (1999). Glycosylphosphatidylinositol biosynthetic enzymes are localized to a stable tubular subcompartment of the endoplasmic reticulum in *Leishmania mexicana*.. EMBO J.

[48] Mullin KA, Foth BJ, Ilgoutz SC, Callaghan JM, Zawadzki JL (2001). Regulated Degradation of an Endoplasmic Reticulum Membrane Protein in a Tubular Lysosome in *Leishmania mexicana*.. Mol Biol Cell.

[49] Docampo R, de Souza W, Miranda K, Rohloff P, Moreno SN (2005). Acidocalcisomes - conserved from bacteria to man.. Nat Rev Microbiol.

[50] Lemercier G, Dutoya S, Luo S, Ruiz FA, Rodrigues CO (2002). A vacuolar-type H+-pyrophosphatase governs maintenance of functional acidocalcisomes and growth of the insect and mammalian forms of *Trypanosoma brucei*.. J Biol Chem.

[51] Kjer-Nielsen L, van Vliet C, Erlich R, Toh BH, Gleeson PA (1999). The Golgi-targeting sequence of the peripheral membrane protein p230.. J Cell Sci.

[52] Gleeson PA, Lock JG, Luke MR, Stow JL (2004). Domains of the TGN: coats, tethers and G proteins.. Traffic.

[53] McConville MJ, Ilgoutz SC, Teasdale RD, Foth BJ, Matthews A (2002). Targeting of the GRIP domain to the trans-Golgi network is conserved from protists to animals.. Eur J Cell Biol.

[54] Vichi A, Payne DM, Pacheco-Rodriguez G, Moss J, Vaughan M (2005). E3 ubiquitin ligase activity of the trifunctional ARD1 (ADP-ribosylation factor domain protein 1).. Proc Natl Acad Sci USA.

[55] Briones MR, Souto RP, Stolf BS, Zingales B (1999). The evolution of two *Trypanosoma cruzi* subgroups inferred from rRNA genes can be correlated with the interchange of American mammalian faunas in the Cenozoic and has implications to pathogenicity and host specificity.. Mol Biochem Parasitol.

[56] Klein S, Franco M, Chardin P, Luton F (2006). Role of the Arf6 GDP/GTP cycle and Arf6 GTPase-activating proteins in actin remodeling and intracellular transport.. J Biol Chem.

[57] Haynes LP, Thomas GM, Burgoyne RD (2005). Interaction of neuronal calcium sensor-1 and ADP-ribosylation factor 1 allows bidirectional control of phosphatidylinositol 4-kinase beta and trans-Golgi network-plasma membrane traffic.. J Biol Chem.

[58] Cuvillier A, Redon F, Antoine J-C, Chardin P, DeVos T (2000). *Ld*ARL-3A, a *Leishmania* promastigote-specific ADP-ribosylation factor-like protein, is essential for flagellum integrity.. J Cell Sci.

[59] Sahin A, Espiau B, Marchand C, Merlin G (2008). Flagellar length depends on *Ld*ARL-3A GTP/GDP Unaltered Cycling in *Leishmania amazonensis*.. Mol Biochem Parasitol.

[60] Barr FA, Short B (2003). Golgins in the structure and dynamics of the Golgi apparatus.. Curr Opin Cell Biol.

[61] Graham TR (2004). Membrane targeting: getting Arl to the Golgi.. Curr Biol.

[62] Setty SR, Strochlic TI, Tong AH, Boone C, Burd CG (2004). Golgi targeting of ARF-like GTPase Arl3p requires its Nalpha-acetylation and the integral membrane protein Sys1p.. Nat Cell Biol.

[63] Lodes MJ, Merlin G, DeVos T, Gosh A, Madhubala R (1995). Increased expression of LD1 genes transcribed by RNA polymerase I in *Leishmania donovani* as a result of duplication into the rRNA gene locus.. Mol Cell Biol.

[64] Espiau B, Lemercier G, Ambit A, Bringaud F, Merlin G (2006). A Soluble Pyrophosphatase, a Key Enzyme for Polyphosphate Metabolism in *Leishmania*.. J Biol Chem.

[65] Prina E, Lang T, Glaichenhaus N, Antoine JC (1996). Presentation of the protective parasite antigen LACK by *Leishmania*-infected macrophages.. J Immunol.

[66] Morgan GW, Allen CL, Jeffries TR, Hollinshead M, Field MC (2001). Developmental and morphological regulation of clathrin-mediated endocytosis in *Trypanosoma brucei*.. J Cell Sci.

[67] Bakalara N, Seyfang A, Baltz T, Davis C (1995). *Trypanosoma brucei* and *Trypanosoma cruzi*: life cycle-regulated protein tyrosine phosphatase activity.. Exp Parasitol.

[68] Campbell RE, Tour O, Palmer AE, Steinbach PA, Baird GS (2002). A monomeric red fluorescent protein.. Proc Natl Acad Sci USA.

[69] Ilgoutz SC, Zawadzki JL, Ralton JE, McConville MJ (1999). Evidence that free GPI glycolipids are essential for growth of *Leishmania mexicana*.. EMBO J.

[70] Robinson DR, Gull K (1994). The configuration of DNA replication sites within the *Trypanosoma brucei* kinetoplast.. J Cell Biol.

[71] Berriman M, Ghedin E, Hertz-Fowler C, Blandin G, Renauld H (2005). The genome of the African trypanosome *Trypanosoma brucei*.. Science.

[72] El Sayed NM, Myler PJ, Bartholomeu DC, Nilsson D, Aggarwal G (2005). The genome sequence of *Trypanosoma cruzi*, etiologic agent of Chagas disease.. Science.

[73] Amor JC, Horton JR, Zhu X, Wang Y, Sullards C (2001). Structures of Yeast ARF2 and ARL1. Distinct roles for the N Terminus in the structure and function of ARF family GTPases.. J Biol Chem.

[74] Panic B, Perisic O, Veprintsev DB, Williams RL, Munro S (2003). Structural basis for Arl1-dependent targeting of homodimeric GRIP domains to the Golgi apparatus.. Mol Cell.

[75] Wu M, Lu L, Hong W, Song H (2004). Structural basis for recruitment of GRIP domain golgin-245 by small GTPase Arl1.. Nat Struct Mol Biol.

