# Supplementary figures and images for: The Leishmania ARL-1 and Golgi Traffic
Source: PLoS One. 2008 Feb 20;3(2):e1620. doi: 10.1371/journal.pone.0001620 (PMC2237903; doi:10.1371/journal.pone.0001620)

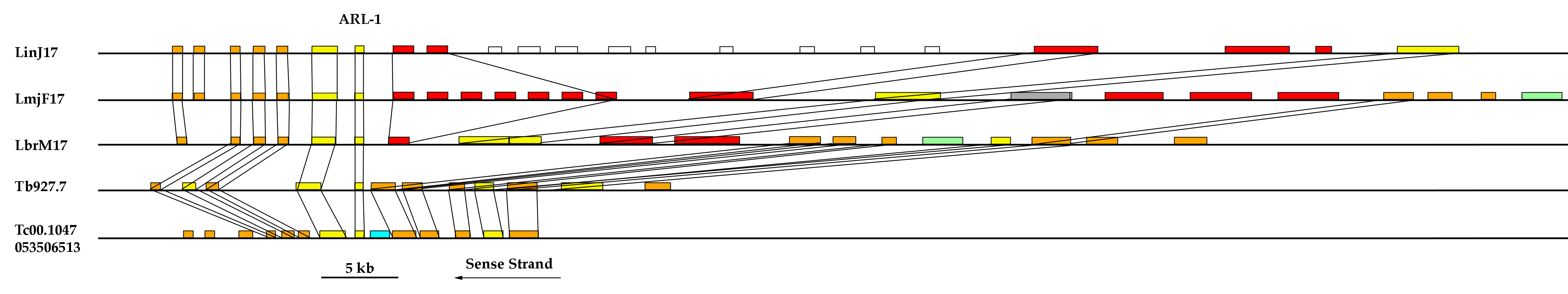

Supplement: Figure S1 — The ARL-1 chromosomal region in trypanosomatids. Genomic map compiled from the Gene DB Parasite Genome database (GeneDB http://www.genedb.org/) [1], [2], [71], [72]. L. major LmARL-1: LmjF17.0070; L. infantum LinfARL-1: LinJ17.0080; L. brasiliensis LbrARL-1: LbrM17_V2.0080; T. brucei TbARL-1: Tb927.7.6230, previously Tb07.2F2.550, annotated as TbARF-3); T. cruzi TcARL-1: two entries: Tc00.1047053506513.60 and Tc00.1047053508919.60. The general organisation is conserved downstream of the ARL-1 ORF, while on the upstream side, several successive insertions and gene duplications occurred in Leishmania (L. brasiliensis>L. major>L. infantum) compared to the trypanosomes. (13.55 MB TIF) [file pone.0001620.s001.tif]

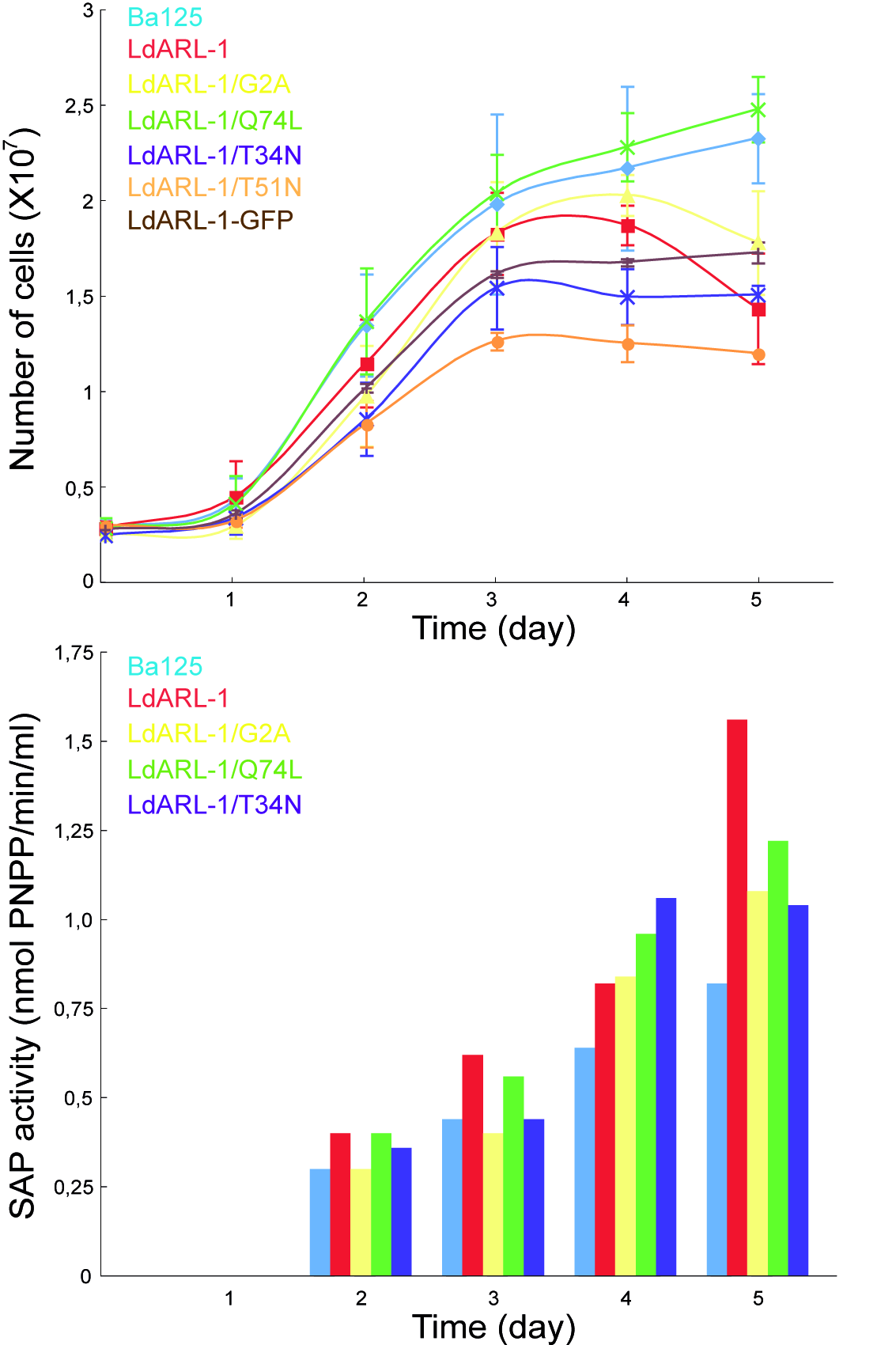

Supplement: Figure S2 — Growth of L. amazonensis strains and SAP activity in the supernatant. Cells. BA125 parental strain, cyan. Promastigotes transformed with pTEX-LdARL-1, brown; pTEX-LdARL-1/G2A, yellow; pTEX-LdARL-1/Q74L, light green; pTEX-LdARL-1/T34N, blue; pTEX-LdARL-1/T51N, orange; pNUS-LdARL-1-GFP, dark green. Top panel: Growth of parental and transformed L. amazonensis promastigotes. Cells were seeded at a density of 2.5 106 cells/ml and counted for the 5 following days; the mean of 2–5 different experiments is indicated with the standard deviation. The maximal cell density appeared a bit lower for some clones, it is not known if these changes are significant. In fact, the level of trangenes expression cannot be accurately controlled; fluorescence microscopy observations revealed that there are variations from cell to cell, from clone to clone or within the same clone after different periods of culture; a more reliable observation might be made with integrated transgene after homologous recombination but it is not known if the experiment is possible at all. Bottom panel: Secreted acid phosphatase (SAP) activity in the culture supernatant. The enzyme activity was determined as in [58] 2–5 days after seeding, and is expressed in nmoles of PNPP hydrolyzed per min and ml of medium. The complete experiment was done once. (9.90 MB TIF) [file pone.0001620.s002.tif]

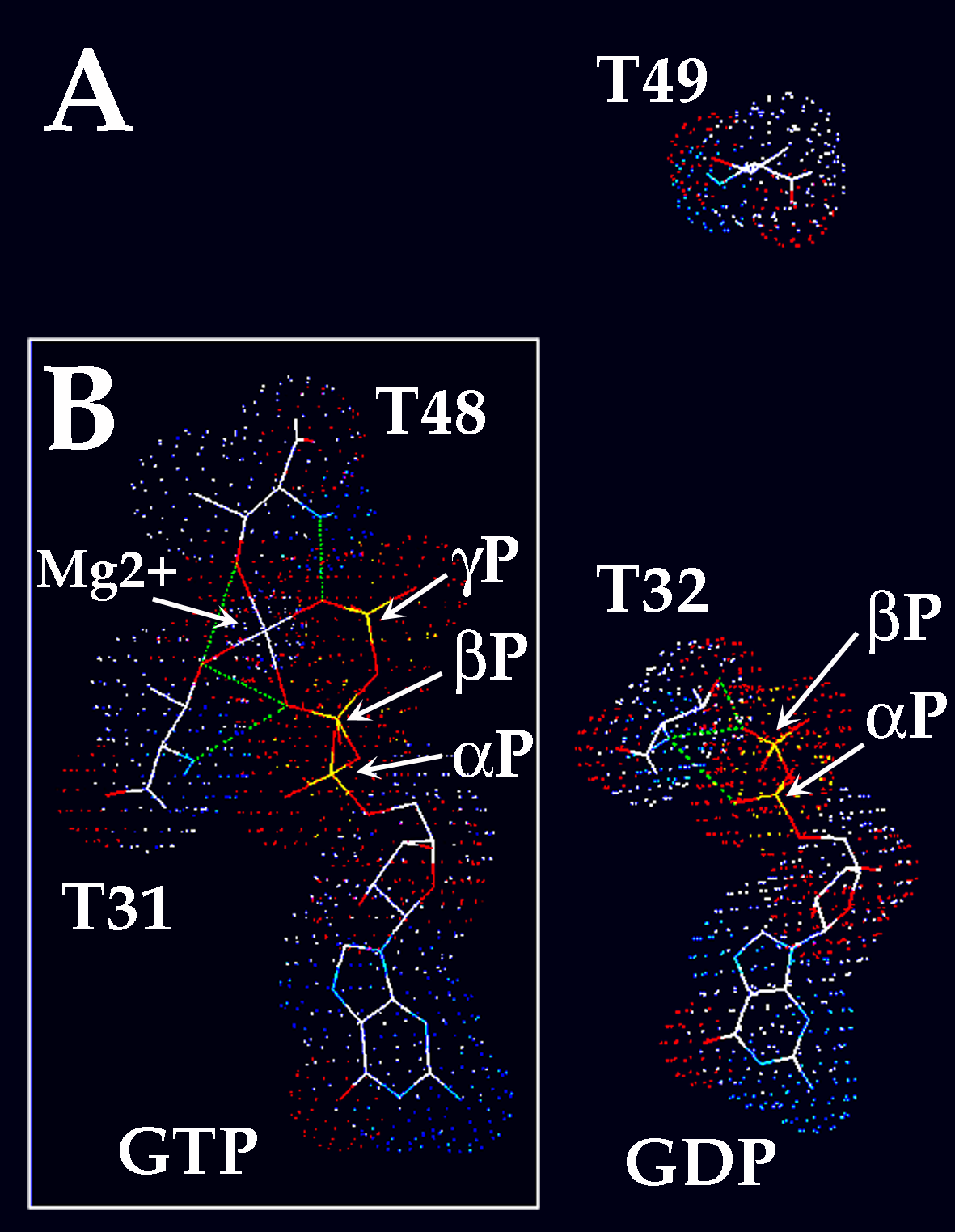

Supplement: Figure S3 — Interactions of Threonines with GTP and GDP. ARF-6 and ARL-1 belong to the same subfamily of ras proteins and their GTP binding sites are well conserved (Fig 1). The structures of two ARL-1 orthologues, the S. cerevisiae ScARL-1/GDP [73] and the human HsARL-1/GTP [74], [75], have been determined. Using the software Swiss-PdbViewer/DeepView for OSX v3.9b1 (http://ca.expasy.org/spdbv/), and the structures of ScARL-1/GDP (Panel A) and HsARL-1/GTP (Panel B), the threonines interacting with the GDP (Panel A) or GTP (Panel B) were selected without modifying their relative positions. Electron density and H-bonds (Green) were emphasised. N = blue, O = red, C = white, H = cyan, P = orange. Similarly to Threonine T27 of HsARF-6, the equivalent threonines T32 of ScARL-1 (Panel A) and T31 of HsARL-1 (Panel B) interact via H-bonds with the α and β phosphorus of GDP (Panel A) and GTP (Panel B). Conversely, like Threonine T44 of HsARF-6, the equivalent Threonine T49 of ScARL-1 (Panel A) does not interact with GDP but the equivalent Threonine T48 of HsARL-1 (Panel B) does interact with GTP. The neighbouring sequences are conserved for all ARL-1 proteins, including LdARL-1 (Fig 1); one might relatively safely predict that the mutant proteins LdARL-1/T34N, ScARL-1/T32N, and HsARL-1/T31N (equivalent to HsARF-6/T27N) lose significantly their affinity not only for GTP but also for GDP, so that these proteins are « empty ». Similarly, the mutations T44N of HsARF-6, T49N of ScARL-1, T48N of HsARL-1 and T51N of LdARL-1 impair the binding of GTP but not GDP, leading to a « GDP-blocked form ». (6.70 MB TIF) [file pone.0001620.s003.tif]
